# Engineered CD40-biosensor-expressing Treg cells as a cell therapy approach for inflammatory diseases

**DOI:** 10.1016/j.ymthe.2026.01.002

**Published:** 2026-01-10

**Authors:** Sebastian Bittner, Lisa Schmidleithner, Brigitte Ruhland, Veronika Hofmann, Philipp Stüve, Frauke Hoffmann, Bernd Echtenacher, Petra Hoffmann, Matthias Edinger, Andreas Beilhack, Nicholas Strieder, Inmaculada Hernandez-Lopez, Michael Rehli, Thomas Hehlgans, Markus Feuerer

**Affiliations:** 1 Leibniz Institute for Immunotherapy (LIT), University Regensburg, 93053 Regensburg, Germany; 2 Chair for Immunology, University Regensburg, 93053 Regensburg, Germany; 3 Department of Internal Medicine III, University Hospital Regensburg, 93053 Regensburg, Germany; 4 Department of Internal Medicine II, Interdisciplinary Center for Clinical Research (IZKF) Laboratory Würzburg, Center for Experimental Molecular Medicine, University Hospital Würzburg, 97078 Würzburg, Germany

**Keywords:** autoimmunity, regulatory T cell, CD40L, GvHD, cell therapy, AIR Treg

## Abstract

Restoring immune tolerance by engineered regulatory T cell (Treg) therapy is a promising strategy to treat patients suffering from autoimmune and inflammatory diseases. However, in many of these conditions, relevant disease-driving antigens are unknown. Therefore, suitable target (auto-)antigens for antigen-specific Treg cell therapy are rarely available. We present a novel artificial immune biosensor for Treg cells that circumvents this limitation by targeting the immune costimulatory protein CD40-ligand (CD40L), transiently expressed by activated T cells. The artificial immune receptor (AIR) comprises a CD40-derived extracellular binding domain, an intracellular costimulatory signaling domain, and a T cell receptor signaling domain of the CD3-ζ-chain. After interaction with its membrane-bound ligand, this synthetic receptor triggers a TCR-like activation program in Treg cells including induction of Treg effector molecules and cell proliferation. In a mouse model of graft-versus-host disease, transfer of CD40-AIR Treg cells significantly improved survival and demonstrated immune control of the alloantigen-reactive T cell compartment. Expression and signaling of the corresponding human CD40-AIR illustrate the potential for translating this concept. Engineering Treg cells with a CD40L-targeting sensor that detects activated T cells presents a promising therapeutic approach for a broad range of T cell-mediated inflammatory diseases.

## Graphical abstract

**Figure:**
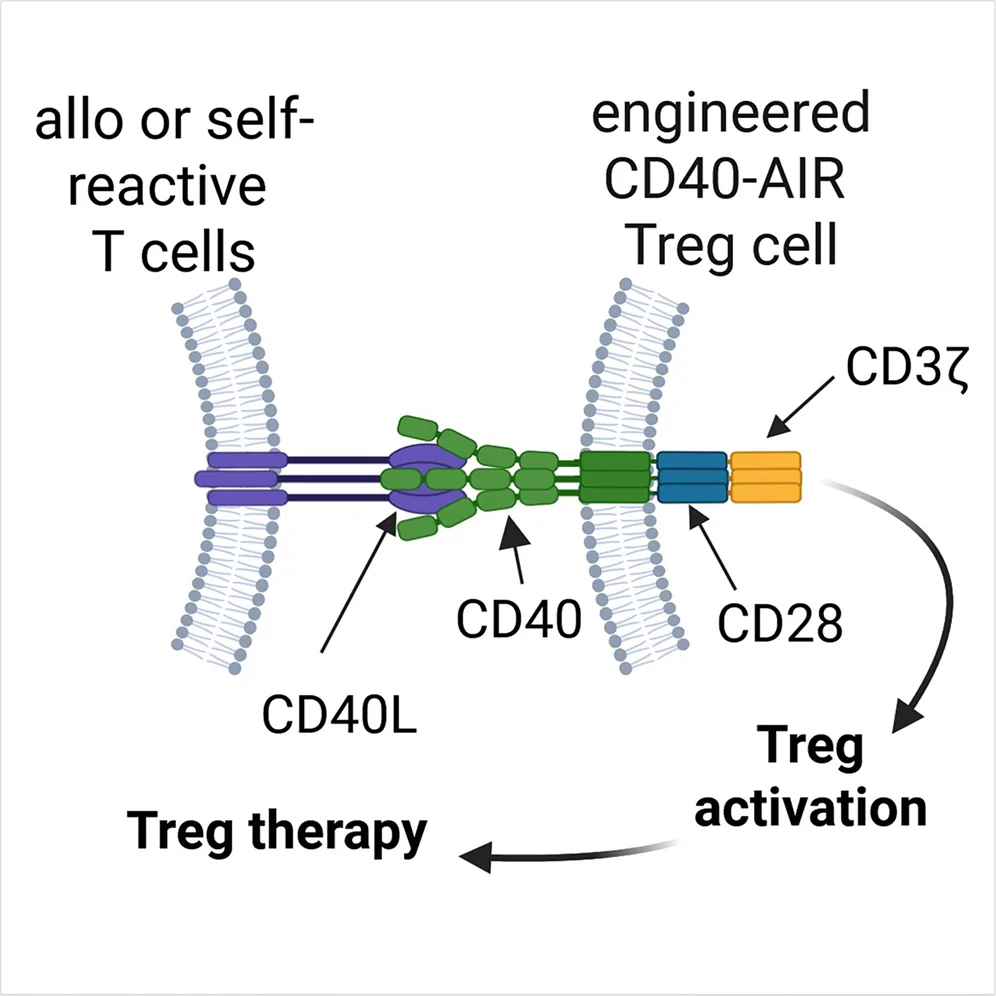

## Teaser

Bittner and colleagues developed novel artificial immune biosensors for Treg cells which trigger Treg cell activation by detecting CD40L on activated T cells, thus eliminating the need to target specific disease-driving antigens. In mouse GvHD disease models, modified Treg cells significantly improved survival, demonstrating broad therapeutic potential for inflammatory conditions.

## Introduction

CD4^+^ regulatory T (Treg) cells, characterized by the expression of the key transcription factor FOXP3, are crucial regulators of the immune system by inducing tolerance against self-antigens and commensal microbiota.^1^ Furthermore, Treg cells located in tissues can undergo a process of tissue adaptation, thereby acquiring the ability to support tissue homeostasis as well as tissue repair.^2^^,^^3^^,^^4^^,^^5^^,^^6^^,^^7^ These unique properties render Treg cells interesting candidates for cell therapy against unwanted immune responses that cause inflammatory and tissue-destructive diseases. The efficacy of Treg cell therapy has been demonstrated in preclinical models of inflammatory diseases such as rheumatoid arthritis, type 1 diabetes, and inflammatory bowel disease (IBD).^8^^,^^9^ Adoptive transfer of Treg cells in organ transplantation can reduce graft rejection and mitigates adverse effects such as graft-versus-host disease (GvHD) after allogeneic hematopoietic cell transplantation (allo-HCT).^10^ Recent findings have demonstrated that antigen-specific Treg cells are more effective in inhibiting autoimmune responses than polyclonal Treg cells making them a more auspicious therapy approach.^9^^,^^11^ Antigen specificity can be artificially introduced into Treg cells by transduction with chimeric antigen receptors (CARs) or recombinant T cell receptors (TCRs). Therefore, the concept of adoptive Treg cell therapy with polyclonal and CAR-engineered cells is currently being intensively investigated in phase I/II clinical trials for various (autoimmune) indications as well as in solid organ and hematopoietic stem cell transplantation.^8^ However, in many autoimmune and inflammatory diseases, disease-driving antigens are unknown and often multiple tissues or organs are affected in an individual patient. To circumvent this problem, we have recently developed novel synthetic receptors for Treg cells, called artificial immune receptors (AIRs), which detect and respond to inflammatory ligands of the tumor necrosis factor superfamily.^12^

CD40 and its ligand CD40L (CD154) play a crucial role not only in the adaptive immune response but also in the pathogenesis of several autoimmune diseases.^13^^,^^14^ CD40L is transiently expressed by activated CD4^+^ and CD8^+^ T cells, while its receptor, CD40, is constitutively expressed by antigen-presenting cells (APCs).^15^^,^^16^ T follicular helper-mediated CD40L interaction with CD40-expressing B cells was shown to be critical for germinal center formation, isotype class switching, and B cell proliferation. In addition, the T cell-APC crosstalk via CD40L/CD40 leads to maturation of dendritic cells and macrophages.^17^^,^^18^^,^^19^ Blockade of the CD40L-CD40 pathway has proven to be an effective treatment for several experimental autoimmune conditions in preclinical models of multiple sclerosis (MS), IBD, and type 1 diabetes.^20^^,^^21^^,^^22^^,^^23^^,^^24^^,^^25^^,^^26^^,^^27^ Meanwhile, this strategy has also been tested in clinical trials for several indications including GvHD, MS, and rheumatic diseases, such as systemic lupus erythematosus (SLE) and myasthenia gravis.^13^^,^^14^^,^^24^^,^^28^^,^^29^

This critical role of the CD40L-CD40 pathway in various inflammatory diseases prompted us to develop a CD40L-specific biosensor, called CD40-AIR for Treg cell therapy. This approach involves engineering of Treg cells to express a CD40-derived extracellular binding domain linked to an intracellular costimulatory domain and the TCR signaling domain of the CD3-ζ-chain. These engineered CD40-AIR Treg cells are capable of sensing activated T cells expressing CD40L and protected animals in a lethal GvHD model by controlling activated alloreactive effector T cells.

## Results

### Engineered CD40-AIR expression and signaling ability

**Figure 1:**
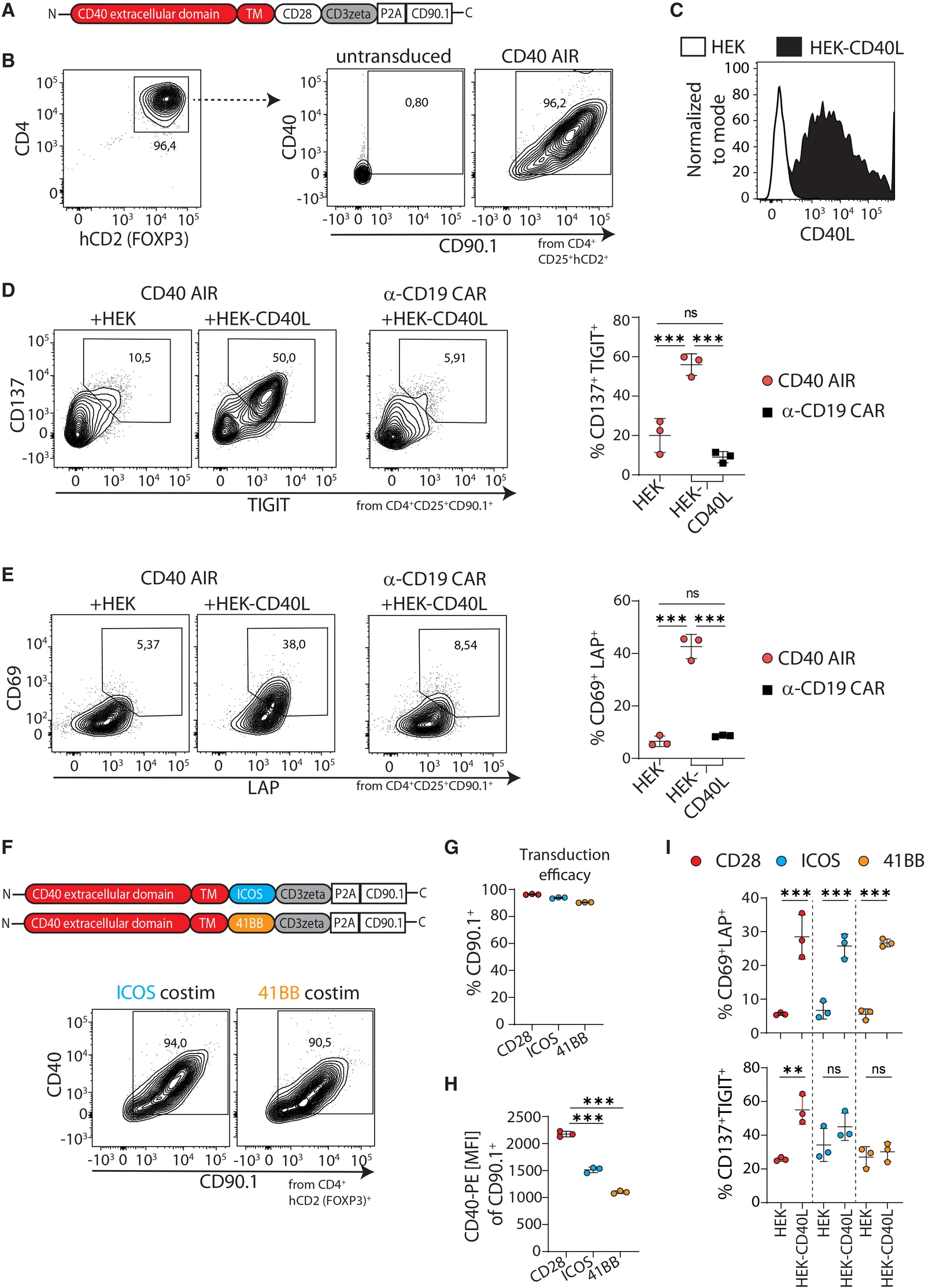
Generation and expression of CD40-AIR (A) Schematic representation of the CD40-AIR construct design. (B) Representative flow cytometric analysis of murine Treg cell cultures 6 days after sorting, showing transduction efficiency (CD90.1 expression) and CD40-AIR surface expression on Treg cells 3 days after transduction. (C) Representative flow cytometric analysis of HEK cells or HEK cells transiently expressing murine CD40L. (D and E) Representative flow cytometric analysis of CD40-AIR or irrelevant α-CD19 CAR-expressing Treg cells that were co-cultured with HEK cells or HEK cells transiently expressing murine CD40L for 18 h (left). Summarized data from three independent experiments (mean of two technical replicates ±SD) are shown on right (*n* = 3, one-way ANOVA). (D) Protein expression of CD137 and TIGIT and (E) CD69 and LAP is shown. (F) Schematic representation of the CD40-AIR construct design with ICOS and 41BB costimulatory domains (upper panel). Representative flow cytometric analysis of murine Treg cells showing CD90.1 and CD40-AIR surface expression on Treg cells (lower panel). (G) Summarized flow cytometric data for CD90.1 expression in Treg cells, transduced with CD28, ICOS, or 41BB containing AIR construct, from three experiments (mean ± SD) are shown (*n* = 3, one-way ANOVA). (H) Mean fluorescence intensity (MFI) for CD40 (PE) of transduced CD90.1^+^ Treg cells from three experiments (mean ± SD) is shown (*n* = 3, one-way ANOVA). (I) Treg cells transduced with CD40-AIR versions containing either CD28 or ICOS or 41BB costimulatory domains were rested for 24 h and then co-cultured with HEK cells or HEK cells expressing CD40L for 18 h and afterward analyzed for CD69 and LAP expression (upper panel) and CD137 and TIGIT expression (lower panel) via flow cytometry (*n* = 3, one-way ANOVA). ∗*p* < 0.05, ∗∗*p* < 0.01, ∗∗∗*p* < 0.001.

As T cells, including Treg cells, lack endogenous CD40 expression, we aimed to engineer a CD40 receptor capable of activating Treg cells upon interaction with CD40L. To this end, we cloned an artificial DNA fragment encoding the extracellular binding as well as the transmembrane domain of murine CD40, followed by the intracellular signaling domain of CD28 and the CD3-ζ-chain into a retroviral vector (schematic representation in Figure 1A). This combination of the CD28 and the CD3-ζ signaling domains has been previously described to induce TCR-like signaling in Treg cells by us and others.^12^^,^^30^ Additionally, the construct included a reporter gene (CD90.1), linked to the AIR sequence via self-cleaving P2A peptide. Treg cells, isolated from spleen and lymph nodes of Foxp3-hCD2 reporter mice, were sorted to high purity via flow cytometry (Figure S1A) and transduced with the retroviral particles during Treg expansion, with α-CD3/CD28 antibody-coupled beads and interleukin-2 (IL-2). Transduced Foxp3^+^ Treg cells were expanded under these conditions and expressed the CD40-AIR receptor as well as CD90.1 on the cell surface (Figure 1B). In co-culture experiments with CD40L-expressing HEK cells (Figure 1C), CD40-AIR Treg cells specifically upregulated the activation markers CD137 (4-1BB) and TIGIT (Figure 1D), CD69, and latency-associated peptide ([LAP], associated with TGF-β1) (Figure 1E). This response required CD40-AIR interaction with CD40L, as HEK cells lacking CD40L did not induce these changes (Figures 1D and 1E) nor did Treg cells equipped with an irrelevant α-CD19 CAR, comprising the same intracellular signaling domains as the CD40-AIR (Figures 1D and 1E). These data demonstrate the functionality of the CD40-AIR construct.

Beyond CD28 co-stimulation, Icos and 4-1BB signaling have also been reported to enhance Treg cell functionality.^31^^,^^32^^,^^33^ Consequently, Icos and 4-1BB signaling domains were previously also introduced into CAR constructs for Treg cells.^34^^,^^35^ Therefore, to test for the best costimulatory signal for the CD40-AIR Treg cells, we generated CD40-AIR variants in which we replaced the CD28 costimulatory domain with either Icos or 4-1BB intracellular domains (schematic representation in Figure 1F). These variants were also expressed on the cell surface of Treg cells (Figure 1F, lower part) with comparable transduction efficacy, as indicated by CD90.1 expression (Figure 1G). Nevertheless, we observed that the mean fluorescence intensity of CD40-AIR expression was lower in the Icos and 4-1BB constructs, compared with the CD28 containing construct (Figures 1H and S1B). Interestingly, co-culture experiments revealed that CD40L-triggered induction of CD69 and LAP was comparable across all three constructs, whereas only the CD28-containing CD40-AIR construct effectively upregulated CD137 and Tigit (Figure 1I, lower part), indicating a somewhat lower activation potential of the Icos and 4-1BB costimulatory domain containing CD40-AIR constructs. To exclude that these differences were caused by the lower expression of the Icos- and 4-1BB-containing CD40-AIR constructs compared with the CD28-containing CD40-AIR construct, we analyzed CD137 and Tigit expression in cell fractions expressing intermediate levels of the CD40-AIR construct. While the protein expression levels of CD137 and Tigit were slightly reduced when analyzing this cell fraction, our initial observation that only the CD28 costimulatory domain led to a significant induction of CD137 and Tigit held true (Figure S1C).

Thus, the newly designed CD40L-binding AIR construct was proved functional and specifically activated Treg cells. The CD28 costimulatory domain seemed a better choice for the CD40-AIR Treg activation, compared with Icos and 4-1BB. This finding aligns with previous studies comparing costimulatory domains for CAR-mediated Treg cell activation.^30^^,^^36^^,^^37^

### CD40-AIR is exclusively activated by membrane-bound CD40L and induces Treg cell proliferation

**Figure 2:**
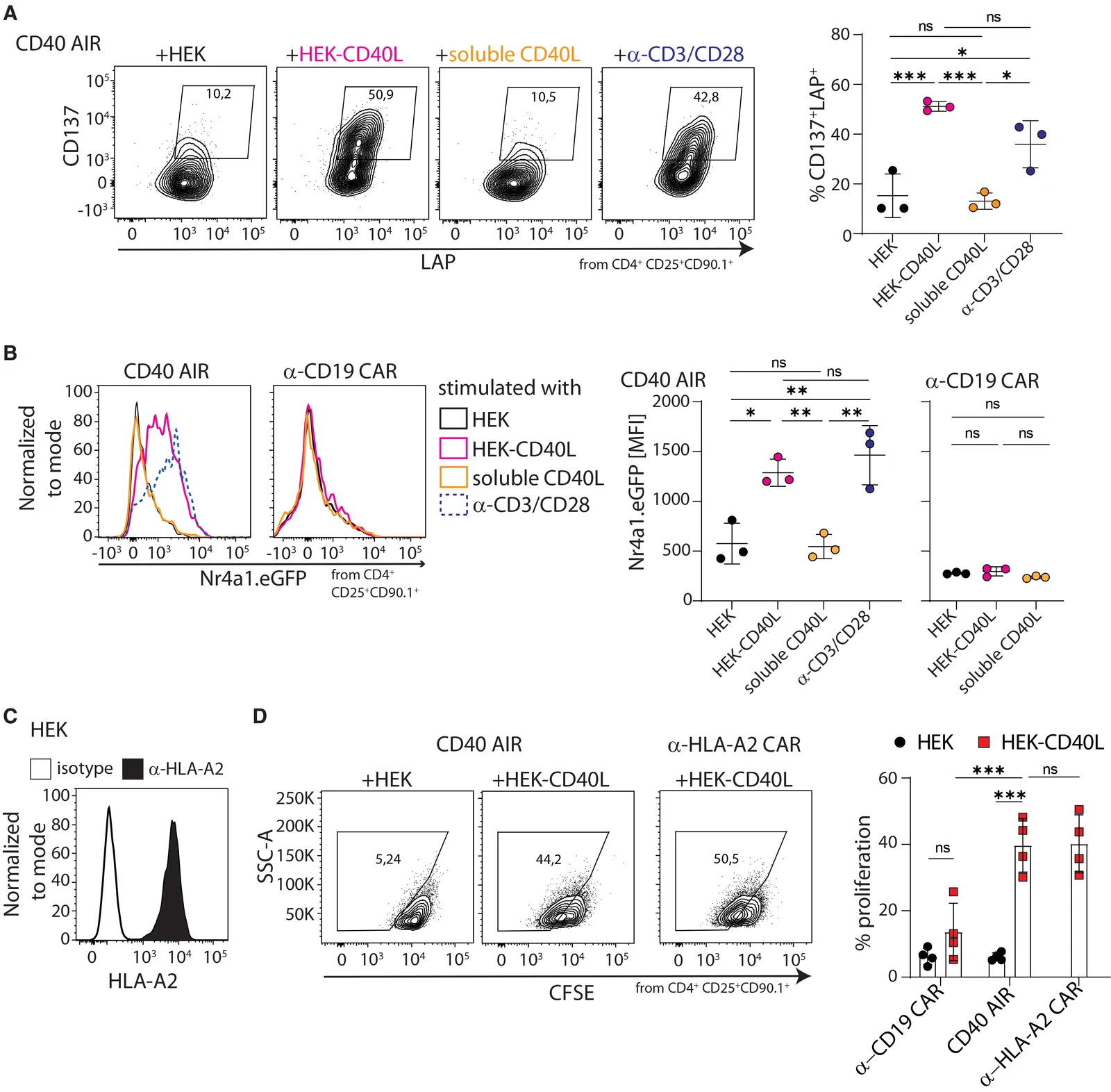
CD40 AIR is activated by membrane-bound CD40L and triggers Nr4a1 upregulation and proliferation (A) CD40-AIR expressing Treg cells were either co-cultured with HEK cells or HEK cells expressing CD40L or stimulated with soluble CD40L or a-CD3/CD28 antibody-coupled beads for 18 h. Afterward, Treg cells were analyzed for CD137 and LAP expression via flow cytometry. Representative flow cytometric data shown on the left and summarized data form three experiments (mean ± SD) on the right (*n* = 3, two-way ANOVA). (B) Treg cells from Nr4a1.eGFP reporter mice were isolated, transduced with CD40-AIR or α-CD19 CAR. After resting for 24 h Treg cells were either co-cultured with HEK cells or HEK cells expressing CD40L or stimulated with soluble CD40L or a-CD3/CD28 antibody-coupled beads for 18 h. Afterward, Treg cells were analyzed for Nr4a1.eGFP expression via flow cytometry. Representative flow cytometric data shown on the left and summarized data form three experiments (mean ± SD) on the right (*n* = 3, two-way ANOVA). (C) HEK cells were analyzed for HLA-A2 expression via flow cytometry. (D) CD40-AIR- or control CAR-expressing Treg cells were rested for 24 h and labeled with CFSE proliferation dye. Engineered and labeled Treg cells were co-cultured with HEK ± CD40L for 72 h in presence of IL-2 and afterward analyzed via flow cytometry for proliferation. Representative dot plots are shown on left. Summarized data from four experiments (mean ± SD) are shown on the right (*n* = 3, two-way ANOVA). ∗*p* < 0.05, ∗∗*p* < 0.01, ∗∗∗*p* < 0.001.

CD40L is expressed by activated T cells as a membrane-bound protein that can be cleaved by metalloproteinases resulting in a soluble, systemically available form of the ligand.^38^ To evaluate whether soluble CD40L could potentially induce systemic activation of the CD40-AIR Treg cells, we compared the effects of membrane-bound CD40L and soluble CD40L on CD40-AIR Treg cell activation. Membrane-bound CD40L effectively activated CD40-AIR-mediated Tregs comparable with polyclonal TCR stimulation with α-CD3/CD28 antibody-coupled beads, while soluble CD40L failed to trigger activation (Figure 2A). This restricted activation of CD40-AIR by a membrane-bound ligand only is a crucial prerequisite ensuring that the engineered Treg cells are locally activated within inflamed tissues as well as in the draining lymph nodes, rather than systemically.

Next, we assessed the induction of the orphan nuclear receptor Nr4a1, which serves as an immediate-early marker of TCR activation and signaling and is also induced by AIR signaling via the CD3-ζ-chain.^12^^,^^39^^,^^40^ Treg cells from transgenic *Nr4a1*.eGFP reporter mice were transduced with the CD40-AIR construct or an irrelevant α-CD19 CAR as control, and co-cultured with CD40L-expressing HEK cells. *Nr4a1*.eGFP expression was induced only in CD40-AIR Treg cells after co-culture with CD40L-expressing HEK cells, but not in α-CD19 CAR-expressing Treg cell controls, with expression levels similar to those following polyclonal TCR activation via α-CD3/CD28-coupled beads (Figure 2B).

Since both TCR and CAR signaling can drive Treg cell proliferation,^30^^,^^41^ we examined the ability of CD40-AIR to induce Treg cell proliferation. Therefore, Treg cells expressing an α-CD19 CAR, a CD40-AIR, or an α-HLA-A2 CAR, all three equipped with the same intracellular signaling domains, were tested for their proliferation capacity. After transduction, CD40-AIR or CAR Treg cells were rested for 24 h without TCR stimulation and then labeled with carboxyfluorescein succinimidyl ester (CFSE). Afterward, Treg cells were co-cultured with HEK cells for 72 h in the presence of IL-2 and analyzed for CFSE dilution. α-HLA-A2 CAR Treg cells, which proliferate in response to the endogenously expressed HLA-A2 antigen on HEK cells,^41^ served as a positive control (Figure 2C). In contrast, α-CD19 CAR Treg cells did not proliferate due to the lack of their corresponding CAR antigen (CD19). However, CD40-AIR Treg cells proliferated in response to CD40L, to a similar extent as the α-HLA-A2 CAR-positive control did, indicating a comparable activation potential of the CD40-AIR and the α-HLA-A2 CAR in Treg cells (Figure 2D).

### CD40-AIR Treg signaling matches CAR Treg signaling

**Figure 3:**
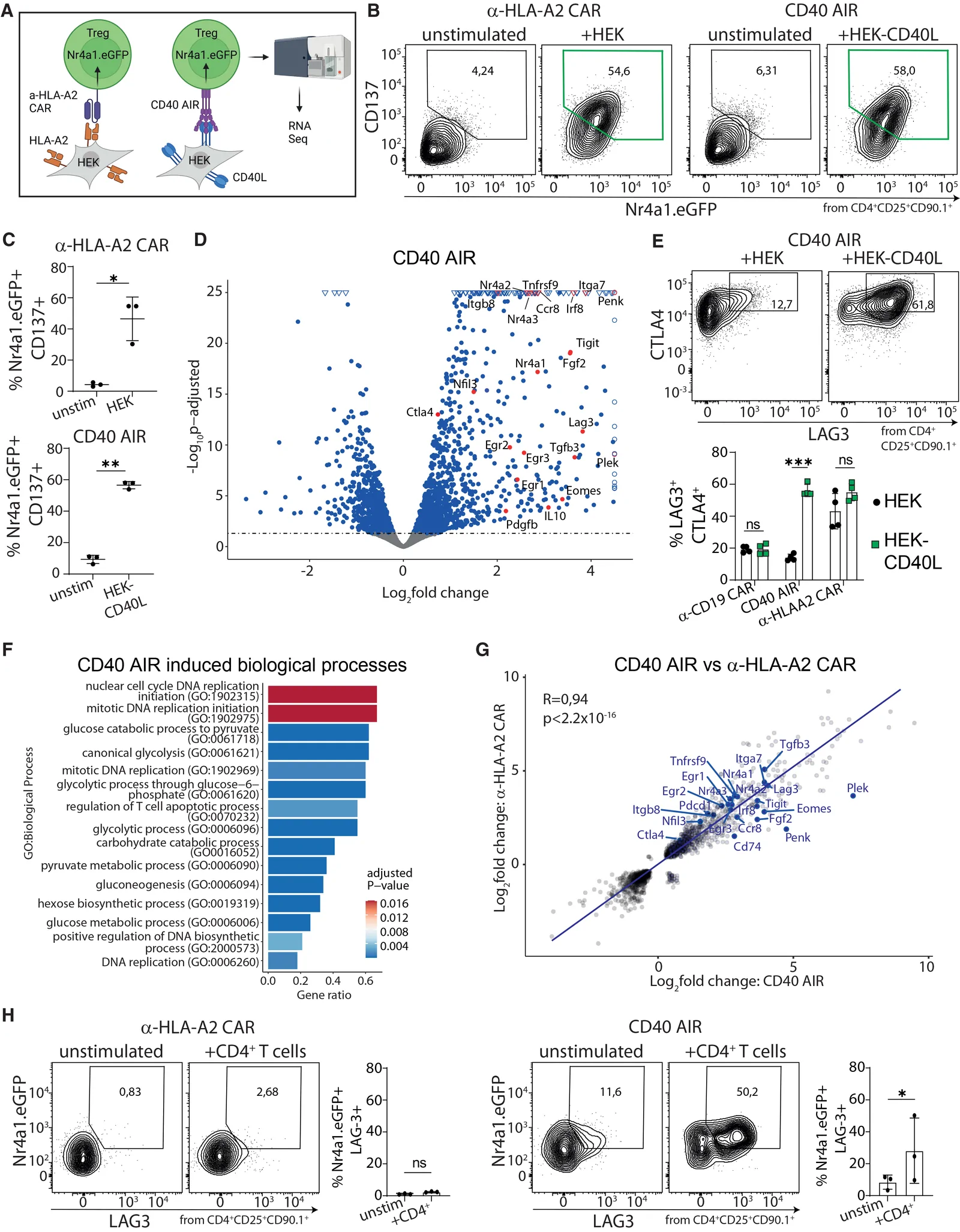
CD40-AIR signaling is comparable with CAR signaling (A) Schematic presentation of the performed co-culture experiment (created with BioRender). (B) CD40-AIR- or a-HLA A2 CAR-expressing Treg cells were rested for 24 h and then co-cultured for 18 h with HEK cells or HEK cells expressing CD40L or left unstimulated. Treg cells were sorted on living CD4^+^CD25^+^CD90.1^+^ (unstimulated) or CD4^+^CD25^+^CD90.1^+^Nr4a1^+^CD137^+^ (AIR/CAR activated). One representative sorting layout is shown. (C) Summarized flow cytometric data of (B) (*n* = 3, Mann-Whitney U). (D) Volcano plot showing differentially expressed transcripts in CD40-AIR-stimulated Treg cells after co-culture with CD40L-expressing HEK cells compared with unstimulated CD40-AIR Treg cells (complete list Table S1). (E) Flow cytometric analysis of CD40-AIR Treg cells after 18 h co-culture with HEK ± CD40L. Protein expression of LAG3 and intracellular CTLA4 is shown in the upper panels. Summarized data of CD40-AIR Treg cells as well as of Treg cells expressing an α-CD19 CAR or α-HLA A2 CAR (lower panel) (*n* = 3, two-way ANOVA). (F) Gene ontology analysis of CD40-AIR-induced transcripts. Selected pathways from top 25 hits of the overrepresentation analysis preformed with enrichR are shown (complete list Table S1). (G) Differentially expressed transcripts of CD40-AIR- and α-HLA A2 CAR-activated Treg cells (versus no stimulation) are compared visualizing their log_2_FoldChanges. The comparison indicates a correlation coefficient R of 0.94 (*p* < 2.2 × 10^−16^). (H) α-HLA A2 CAR-expressing (left) or CD40-AIR-expressing (right) Treg cells (isolated and generated from Nr4a1.eGFP reporter mice) were rested for 24 h and then co-cultured for 18 h with unstimulated or previously PMA/ionomycin-stimulated CD4^+^ T cells. Protein expression of Nr4a1.eGFP and LAG3 was measured via flow cytometry and data from three experiments are summarized (*n* = 3, paired *t* test). ∗*p* < 0.05, ∗∗*p* < 0.01, ∗∗∗*p* < 0.001.

To further explore similarities between CD40-AIR and CAR signaling in Treg cells, we directly compared CD40-AIR Treg cells with α-HLA-A2 CAR Treg cells, whose human counterparts are currently tested for preventing graft rejection in clinical phase I trials in patients undergoing solid organ transplantation.^42^ To this end, we prepared CD40-AIR and α-HLA-A2 CAR-expressing Treg cells from *Nr4a1*.eGFP reporter mice and co-cultured them with HEK cells expressing CD40L or HEK cells only expressing the endogenous HLA-A2 antigen. Following overnight co-culture, we sorted Treg cells via FACS into AIR- or CAR-activated and non-activated cell populations for RNA-seq (Figure 3A). To this end, we sorted unstimulated CD4^+^CD25^+^CD90.1^+^ Treg cells of both groups, as well as activated *Nr4a1*.eGFP^+^CD137^+^CD90.1^+^ CD40-AIR and α-HLA-A2 CAR Treg cells (Figure 3B). After activation of CD40-AIR and α-HLA-A2 CAR Treg cells, with their respective antigens, *Nr4a1*.eGFP and CD137 expression was induced in a majority of transduced Treg cells (Figures 3B and 3C). Compared with unstimulated CD40-AIR-expressing Treg cells, CD40-AIR stimulation by CD40L triggered expression changes of more than 2,500 genes in Treg cells (Figure 3D; Table S1). These included TCR-downstream target genes like *Egr1*, *Egr2*, *Egr3*, *Nr4a1*, *Nr4a2*, and *Nr4a3*, as well as other transcription factors like *Irf8* and *Nfil3*. Several important Treg cell functional molecules such as *Tnfrsf9*, *Fgf2*, *Pdgfβ*, *Ccr8*, *Ctla4*, *Tigit*, and *Lag3* were additionally upregulated (Figures 3D and S2B). The induction of many of these genes was also found in α-HLA-A2 CAR-activated Treg cells (Figures S2A and S2B; Table S2). We could verify the induction of the Treg effector molecule LAG3 on protein level via FACS, for both CD40-AIR as well as α-HLA-A2 CAR-activated Treg cells (Figures 3E and S2B), but not in α-CD19 CAR Treg cells used as a negative control (Figures 3E and S2C). Expanded, Treg cells expressed high levels of Ctla4 already before restimulation (Figures 3E, S2B, and S2C). A further significant increase in protein level was only detected in response to CD40L in CD40-AIR Treg cells, but not in α-CD19 CAR or α-HLA-A2 CAR Treg cells (Figure S2D).

Analysis of gene ontology using the RNA-seq data from CD40-AIR-activated Treg cells revealed that AIR signaling enhances multiple pathways related to cellular glycolysis and pathways associated with cell proliferation, e.g., nuclear cell-cycle DNA replication initiation (GO:1902315) and mitotic DNA replication (GO:1902969) (Figure 3F).

To provide a more in-depth analysis of the similarities between CD40-AIR- and α-HLA-A2 CAR-activated Treg cells, we conducted a detailed comparison of the RNA-seq data of CD40-AIR Treg cells activated through CD40L and α-HLA-A2 CAR Treg cells activated via the HLA-A2 antigen. Plotting differentially regulated genes of CD40 AIR-activated (versus unstimulated CD40-AIR Treg cells) against α-HLA-A2 CAR-activated Treg cells (versus unstimulated α-HLA-A2 CAR Treg cells) (Figure 3G) revealed a strong correlation between both activation groups (R value 0.94), indicating that the global intracellular signaling induced by both synthetic receptors, AIR and CAR, is nearly identical.

To study if CD40L expressed by primary CD4^+^ T cells can induce CD40-AIR-mediated activation of Treg cells, we pre-stimulated CD4^+^ T cells with PMA/ionomycin for 3 h to induce CD40L expression (Figure S2E). After careful removal of any traces of PMA/ionomycin, we used these CD40L-expressing CD4^+^ T cells in co-culture experiments. CD40-AIR Treg cells responded to CD40L expressing CD4^+^ T cells, as exemplified by the upregulation of *Nr4a1*.eGFP and LAG3 (Figure 3H). Conversely, the irrelevant α-HLA-A2 CAR Treg cells did not respond to activated CD4^+^ T cells under the same culture conditions since murine CD4^+^ T cells do not express HLA-A2 (Figure 3H).

In addition to enabling antigen-specific cellular activation, CARs allow Treg cells to remove antigens and parts of the cell membrane in a process called trogocytosis.^43^ This process allows CAR Treg cells to remove costimulatory molecules from other cell types, thereby suppressing unwanted immune cell activation, e.g., during the development of GvHD. Therefore, we tested whether CD40-AIR Treg cells could remove CD40L from the surface of HEK cells. Indeed, we detected the transfer of CD40L protein from HEK cells stained with a labeled α-CD40L antibody before co-culture, specifically to CD40-AIR Treg cells and not to control Treg cells that express only the truncated receptor version, indicating trogocytosis by the CD40-AIR (Figure S2F).

In summary, CD40-AIR Treg signaling closely mirrors CAR Treg signaling, with both synthetic receptors triggering robust Treg cell activation and proliferation. Moreover, CD40-AIR Treg cells can recognize and respond to CD40L expressed by activated T cells, further underscoring the therapeutic potential of CD40-AIR Treg cells.

### CD40-AIR Treg cells provide protection against GvHD

**Figure 4:**
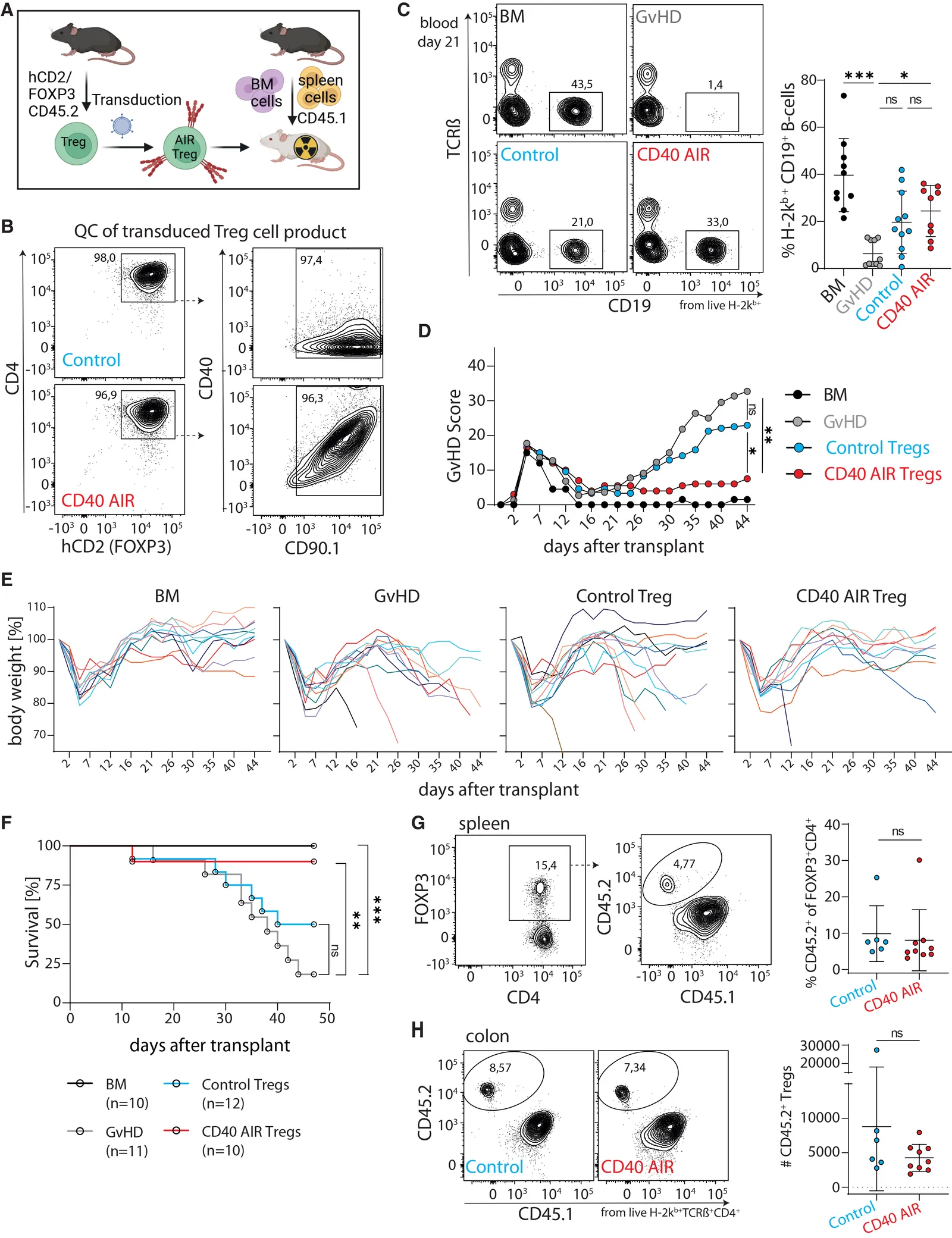
CD40-AIR Treg cells ameliorate GvHD pathology in mice (A) Schematic overview (created with BioRender) of allo-HCT model (complete major histocompatibility complex mismatch, C57/BL6 into BALB/c) to study graft-versus-host disease (GvHD). (B) Flow cytometric analysis of engineered Treg cells before transplantation into mice. (C) On day 21, blood samples from transplanted animals were taken to check for donor-derived (H-2Kb^+^) CD19^+^ B cells via flow cytometry. Graph contains datasets from two independent experiments (Kruskal-Wallis test, *n* = 9–11). (D) Mean GvHD score per group is shown. Area under the curve (AUC) (days 14–44) was calculated for individual animals and then groups were compared (*n* = 10–12, Mann-Whitney U). (E) Body weight shown for each individual animal. Graphs contain datasets from two independent experiments. (F) Kaplan-Meier curve with survival of transplanted BALB/c mice. Graph contains datasets from two independent experiments (log rank test, *n* = 10–12). (G) Representative flow cytometric analysis of cells isolated from the spleen of a mouse receiving CD40-AIR Treg cells. Frequencies of engineered transferred (CD45.2) CD40-AIR or control Treg cells within the Treg compartment at day 47 were compared (control *n* = 6, CD40-AIR *n* = 9; Mann-Whitney U). (H) Representative flow cytometric analysis of CD4^+^ T cells isolated from colon of a CD40-AIR Treg cell-treated mouse (left). Absolute numbers of transferred (CD45.2) CD40-AIR or control Treg cells re-isolated from colon at day 47 were counted (control *n* = 6, CD40-AIR *n* = 9; Mann-Whitney U). ∗*p* < 0.05, ∗∗*p* < 0.01, ∗∗∗*p* < 0.001.

Patients undergoing allo-HCT can develop severe and sometimes fatal GvHD elicited by alloreactive donor T cells present in the graft. Administration of Treg cell products have shown promising results in preclinical studies for the prevention of GvHD.^44^^,^^45^^,^^46^ Given that CD40L expression on alloreactive CD4^+^ donor T cells has been identified as a critical factor in GvHD onset and severity^47^^,^^48^ and that antibody-mediated CD40L blockade can mitigate GvHD development,^49^^,^^50^ we evaluated the therapeutic potential of CD40-AIR Treg cells for the treatment of GvHD in a complete major histocompatibility complex mismatch model of allo-HCT (Figure 4A). Treg cells, derived from C57BL/6 mice (Foxp3-hCD2 reporter, congenic CD45.2), were transduced with either the CD40-AIR or a truncated variant of CD40-AIR, lacking the extracellular domain, serving as the control. These engineered Treg cells were transplanted into myeloablatively irradiated BALB/c mice along with bone marrow (BM) and spleen cells, containing a subset of alloreactive T cells. Prior to adoptive transfer, transduced Treg cells showed a high Treg cell purity (>96% hCD2/FOXP3 positive) and transduction efficiency (>95%) (Figure 4B). On day 21 after transplantation, peripheral blood samples were analyzed for recovery of the B cell compartment after irradiation. Low B cell counts are indicative of an impaired immune reconstitution after irradiation due to ongoing GvHD and, in the case of Treg cell co-transfer, an indication of suboptimal protection.^45^^,^^51^ Mice receiving only BM cells (BM group) successfully reconstituted their B cell compartment while those also receiving splenocytes (GvHD group) did not (Figure 4C). Both control Treg cells and CD40-AIR Treg cells supported B cell recovery, but only the CD40-AIR Treg group reached statistically significant improvement compared with the GvHD group (Figure 4C). Interestingly, similar frequencies of circulating transferred control and CD40-AIR Treg cells (congenic CD45.2) were detected in the blood, indicating comparable Treg engraftment and persistence (Figure S3A). Clinical GvHD scoring, which evaluated body weight, fur appearance, activity, and other parameters, revealed a significantly lower GvHD score in the CD40-AIR Treg treatment group (Figure 4D; GvHD scoring described in materials and methods), including less-pronounced weight loss (Figure 4E), compared with both the GvHD group and the control Treg cell group. Most notably, overall survival was markedly improved in the CD40-AIR Treg-treated group (9/10 mice) as compared with the control Treg group (6/12) and the GvHD group (2/11) (Figure 4F).

On day 47 after allo-HCT, both CD40-AIR Treg cells and control Treg cells were detected in the Foxp3^+^ Treg compartment at comparable frequencies within the spleens of surviving mice, confirming their persistence and stable Treg phenotype (Figure 4G). Additionally, both groups of Treg cells migrated to the colon, a major target organ in GvHD, where they maintained a stable presence (Figure 4H). Klrg1, a marker for differentiation into the tissue-Treg like phenotype,^2^ was detected in Treg cells isolated from the colon at the end of the experiment (Figure S3B). This finding indicated differentiation of CD40-AIR and control Treg cells into the protective tissue-Treg phenotype, as we have shown before for LTBR-AIR and non-transduced *in-vitro-*expanded donor Treg cells.^12^^,^^46^ Furthermore, reduced B cell numbers in mice that were euthanized due to high GvHD score in the control Treg cell group contrasted with normal B cell numbers in surviving control and CD40-AIR Treg groups (Figure S3C), indicating successful Treg-mediated immune tolerance in the surviving animals.

In summary, CD40-AIR-expressing Treg cells demonstrated functionality *in vivo* and provided superior protection against fatal GvHD compared with control Treg cells. These findings confirm the effectiveness of the CD40-AIR sensor concept *in vivo*.

### CD40-AIR Treg cells inhibit function, differentiation, and cell cycle of donor T cells

**Figure 5:**
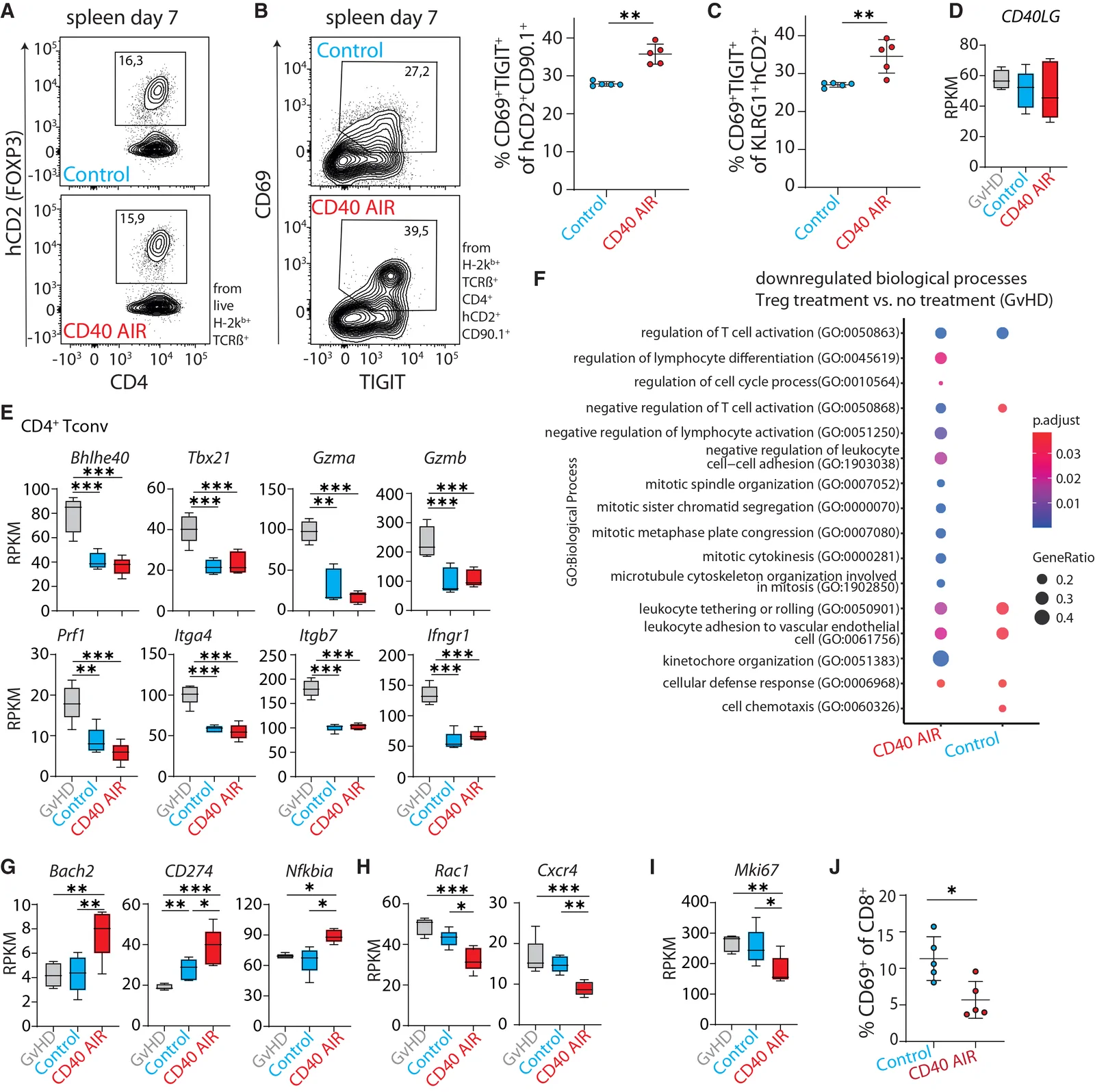
CD40-AIR Treg cells suppress cell cycle and function of donor CD4^+^ T cells (A) Representative flow cytometric analysis of transferred hCD2^+^ Treg cells in cells isolated from spleen of a CD40-AIR or a control Treg cell receiving mouse 7 days after transplantation. (B) Representative flow cytometric analysis of transferred hCD2^+^CD90.1^+^ Treg cells isolated from spleen of CD40-AIR- or control Treg cell-treated mouse 7 days after transplantation. Protein expression for CD69 and TIGIT is shown on the left, summarized data are shown on the right (control *n* = 5, CD40-AIR *n* = 5; Mann-Whitney U). (C) Summarized data showing frequencies of CD69^+^TIGIT^+^ double-positive cells of transferred hCD2^+^KLRG1^+^CD90.1^+^ Treg cells, isolated from spleens of CD40-AIR- or control Treg cell-treated mice (control *n* = 5, CD40-AIR *n* = 5; Mann-Whitney U). (D and E) 7 days after allo-HCT, donor-derived (H-2k^b+^CD45.1^+^) TCR^+^hCD2^−^CD4^+^ T cells were FACS sorted and RNA sequencing was performed. RNA expression data from T cells isolated from mice receiving no treatment, control Treg cells, or CD40-AIR Treg cells are shown (GvHD *n* = 5, control *n* = 5, CD40-AIR *n* = 5; Deseq2). (F) Downregulated pathways in CD4^+^ T cells from CD40-AIR- or control Treg-treated mice individually compared with no treatment (GvHD group). Significant GO Biological Process terms from the 2021 enrichR database are displayed (adjusted *p* < 0.05). All terms from the control Treg treatment are shown, while additional biologically relevant pathways identified in the CD40-AIR group were manually selected. Complete lists of pathways are provided in Tables S3 and S4. (G and H) RNA expression for *Bach2*, *CD274*, *Nfkbia*, *Rac1*, and *Cxcr4* in T cells isolated from mice receiving no treatment, control Treg cells, or CD40-AIR Treg cells are shown (GvHD *n* = 5, control *n* = 5, CD40-AIR *n* = 5; Deseq2). (I) RNA expression for *Mki67* in T cells isolated from mice receiving no treatment, Control Treg cells or CD40-AIR Treg cells are shown (GvHD *n* = 5, control *n* = 5, CD40-AIR *n* = 5; Deseq2). (J) Summarized data showing frequencies of CD69^+^-positive CD8+ cells isolated from spleens of mice treated with CD40-AIR or control Treg cells, 7 days after transfer (control *n* = 5, CD40-AIR *n* = 5; Mann-Whitney U).

Next, we explored the mechanisms behind the protective effects of CD40-AIR Treg cells. Donor-derived conventional CD4^+^ T cells express high levels of CD40L during the early phase of GvHD due to their TCR-mediated activation by host alloantigens.^49^^,^^52^ To investigate this, we performed a short-term GvHD experiment and analyzed the spleens of mice 7 days after allo-HCT. Flow cytometry revealed that CD40-AIR and control Treg cells were present at similar frequencies at this early disease stage, with CD40-AIR Treg cells specifically expressing the CD40 sensor on their surface (Figures 5A, S4A, and S4B). Notably, CD40-AIR Treg cells exhibited higher levels of activation markers CD69 and Tigit compared with control Treg cells at this time point, indicating more robust activation through CD40-AIR signaling (Figure 5B). This elevated activation persisted even in the subset of already tissue-Treg-like differentiated engineered Treg cells (Klrg1^+^), highlighting the profound activation via the CD40-AIR sensor (Figures 5C and S4C).

Given the critical role of CD4^+^ conventional T cells (Tconv) in driving GvHD, we focused on how CD40-AIR Treg cells affect donor-derived CD4^+^ Tconv cells. Therefore, we re-isolated and FACS-purified donor-derived CD4^+^ Tconv from host spleens 7 days after allo-HCT and performed bulk RNA-seq. Unbiased principal-component analysis revealed profound gene expression changes between CD4^+^ Tconv from untreated GvHD mice and those treated with Treg cells, with distinct differences between CD40-AIR and control Treg cell treatments (Figure S4D). The CD40-AIR target molecule, CD40L, was highly expressed in donor CD4^+^ Tconv (Figure 5D). Overall, RNA profiles revealed strong effects of both Treg treatment groups on donor CD4^+^ Tconv cells regarding differentiation, function, and migratory/adhesion molecules. For example, the transcription factors *Bhlhe40* and *Tbx21* (T-bet), both critical for T cell-mediated GvHD pathogenesis,^53^^,^^54^ were significantly reduced upon both Treg cell treatments (Figure 5E). Also, T cell effector molecules like cytolytic granzymes (*Gzma* and *Gzmb*) and perforin (*Prf1*), with described relevance for GvHD development^55^^,^^56^ as well as integrin α4β7 (*Itga4*/*Itgb7*), essential for gut homing of alloreactive T cells,^57^^,^^58^ were significantly decreased compared with the no Treg cell GvHD group (Figure 5E). However, important molecules for graft-versus-leukemia effects, such as *Tnf*, *Ifng*, and *Fasl*,^59^^,^^60^ remained unaffected by both Treg treatments (Figure S4E), in line with recent findings.^60^ Treg cell transfer suppressed expression of other β-integrins (*Itgb1* and *Itgb2*), important for cell adhesion, leukocyte diapedesis, and binding to APCs (Figure S4E). Both Treg cell therapies also downregulated chemokine receptors *Ccr2*, *Cxcr6*, and *Cxcr3* (Figure S4E), all relevant for T cell trafficking to GvHD target organs like lung, skin, and intestine.^61^ Additionally, the expression of certain cytokine receptors was also reduced compared with the GvHD group, as shown for *Ifngr1* (Figure 5E), *Il18r1*, *Il18rap*, *IL2rb*, and *Il7r* (Figure S4E).

In addition to analyzing individual genes, we used gene ontology analysis to compare under-represented biological processes in the donor-derived CD4^+^ T cells isolated from the control or the CD40-AIR Treg-treated group versus the non-treated GvHD group. This analysis showed pathways like leukocyte tethering or rolling (GO:0050901) and regulation of T cell activation (GO:0050863) to be significantly downregulated by both Treg treatments (Figure 5F; Tables S3 and S4); however, particular processes associated with cell division and proliferation such as mitotic spindle organization (GO:0007052) or mitotic cytokinesis (GO:0000281) were significantly impaired only by CD40-AIR Treg but not by the control Treg treatment (Figure 5F).

To further investigate the differences between the CD40-AIR Treg and the control Treg treatment groups, donor-derived CD4^+^ Tconv cells from both Treg treatments were directly compared. We identified several differentially expressed genes and pathways with relevance for T cell function in GvHD pathology. For example, general T cell inhibitory genes, like transcription factor BTB and CNC homolog 2 (*Bach2*),^62^ programmed cell death 1 ligand 1 (Pdl1/*CD274*), and NF-kappa-B inhibitor alpha (*Nfkbia*-encoding IkBa) were significantly upregulated upon CD40-AIR Treg cell treatment (Figure 5G).^62^^,^^63^^,^^64^^,^^65^ Expression of actin-rearranging GTPase *Rac1*, critical for T cell migration and adhesion,^66^ and *Cxcr4*, important for T cell homing to and damage of skin and lung during GvHD,^67^ were significantly downregulated upon CD40-AIR Treg treatment compared with the control or the no treatment GvHD group (Figure 5H). Expression of the transcriptional regulator DNA-binding protein inhibitor 2 (*Id2*) was also stronger reduced in the CD40-AIR-treated group (Figure S4F), which could be of functional relevance, since *Id2* deficiency in alloreactive T cells was shown to abrogate their capability to elicit GvHD.^68^

Besides these findings, donor effector CD4^+^ T cells from CD40-AIR Treg-treated mice might exhibit increased susceptibility to apoptosis due to downregulation of the apoptosis repressor *E2f2*, which in turn results in upregulation of the death receptor *Fas* (Figure S4G), a well-described cell death mechanism in activated T cells.^69^ Additionally, *Birc5* (survivin) another inhibitor of the apoptosis gene was selectively reduced in alloreactive donor CD4^+^ Tconv cells upon CD40-AIR Treg cell therapy (Figure S4G).

When comparing GO biological processes in donor CD4^+^ T cells isolated from CD40-AIR- and control Treg cell-treated mice, we detected downregulated processes that were associated with cell-cycle progression and mitosis such as mitotic cell-cycle phase transition (GO:00444772) or mitotic nuclear membrane disassembly (GO:0044772) specifically in T cells isolated from mice treated with CD40-AIR Treg cells (Figure S4H; Table S5). Examples of downregulated genes in the CD40-AIR treatment were cyclins and cyclin-dependent kinases, e.g., *Cdk1*, from the GO term mitotic cell-cycle phase transition (Figure S4I). A similar downregulation of cell-cycle-associated pathways annotated in the Reactome database was specifically observed with CD40-AIR treatment when comparing the two Treg cell treatments (Figures S4J and S4K). In support of these observations, the expression of the well-established proliferation marker *Mki67* (encoding Ki-67) was significantly inhibited upon CD40-AIR Treg cell treatment compared with control Treg or no treatment GvHD groups (Figure 5I). In addition to these changes in donor CD4^+^ Tconv cells, CD40-AIR Treg treatment also reduced donor CD8^+^ T cell activation more effectively than control Treg cells, indicating superior control over both CD4^+^ and CD8^+^ donor T cells (Figure 5J).

In summary, while both Treg treatments modulate the differentiation, function, migration, and adhesion of donor CD4^+^ Tconv and donor CD8^+^ T cells, CD40L-activated CD40-AIR Treg cells provide stronger control over alloreactive T cells by more effectively inhibiting cycle progression and potentially inducing apoptosis. These combined mechanisms enhance the efficacy of AIR Treg cell therapy.

### Engineered human CD40-AIR Treg cells detect and respond to CD40L

**Figure 6:**
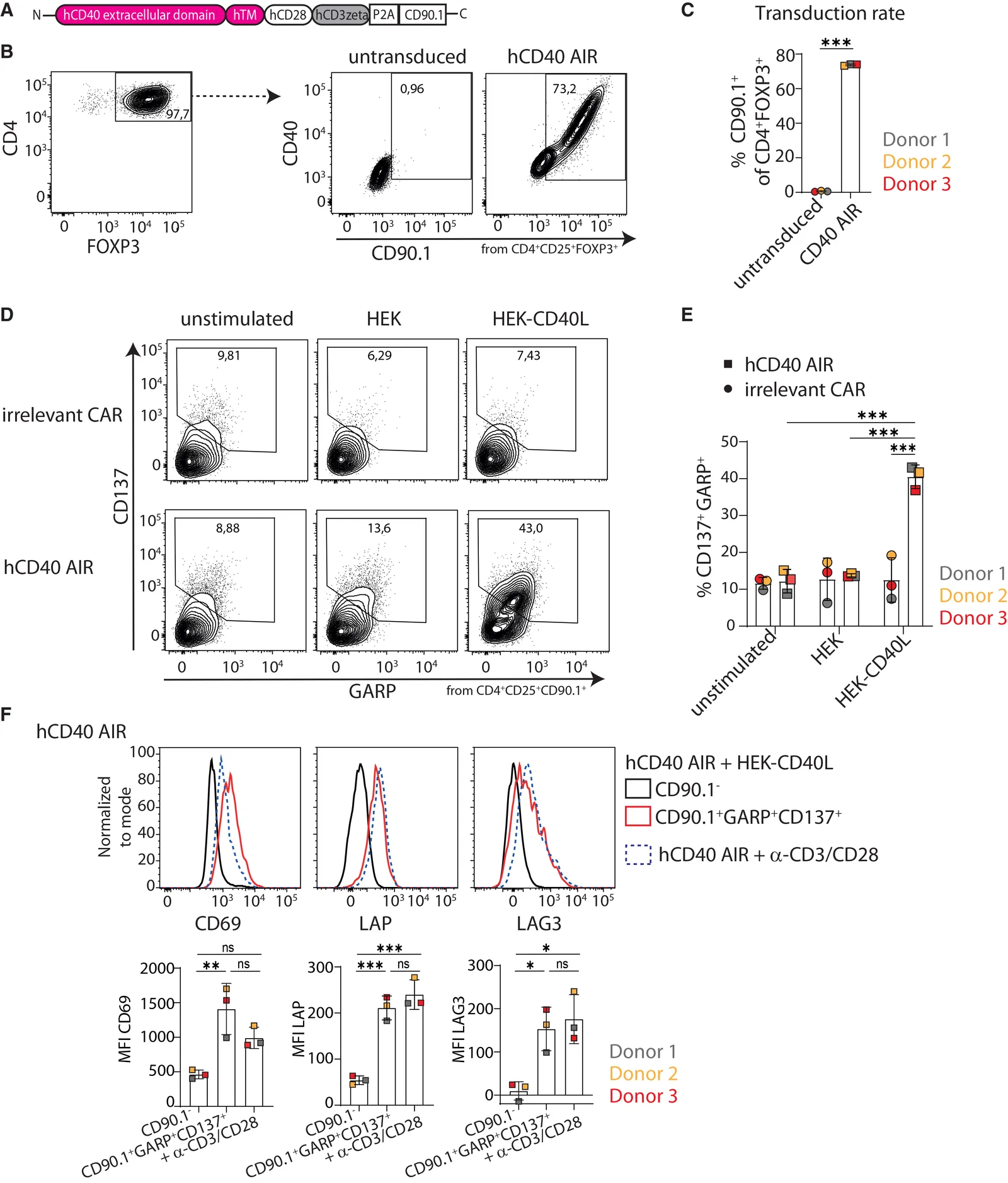
Human CD40-AIR expression and signaling (A) Schematic representation of the human CD40-AIR construct design. (B) Representative flow cytometric analysis of human Treg cell cultures 6 days after sorting, showing transduction efficiency (CD90.1 expression) and CD40-AIR surface expression on Treg cells 4 days after transduction. (C) Summarized data for the transduction efficacy for three different donors. (D) Flow cytometric analysis of Treg cells expressing hCD40-AIR or an irrelevant CAR (α-CEA, carcinoembryonic antigen) after 18 h without stimulation or in co-culture with HEK cells or HEK cells expressing hCD40L protein on the surface. Representative flow cytometric data for protein expression of GARP and CD137 are shown. Data from one donor out of three are shown. (E) Summarized data of three donors from experiment described in (D) (*n* = 3, two-way ANOVA). (F) Flow cytometric analysis of CD90.1^–^ and CD90.1^+^ hCD40-AIR Treg cells after 18 h of co-culture with hCD40L-expressing HEK cells or after α-CD3/CD28 stimulation. Protein expression for CD69, LAP and LAG3, for one donor out of three is shown in the upper panel, summarized data for all three donors are shown in the lower panel (*n* = 3, one-way ANOVA). ∗*p* < 0.05, ∗∗*p* < 0.01, ∗∗∗*p* < 0.001.

To demonstrate that the CD40-AIR concept can be applied to human Treg cells, we developed a human version of the CD40-AIR (hCD40-AIR). This construct mirrors the mouse biosensor construct, incorporating the human CD40 extracellular and transmembrane domains, along with intracellular signaling domains from human CD28 and CD3-ζ (Figure 6A). To test for functionality, we FACS-sorted human Treg cells for TCR-β^+^CD4^+^CD25^+^CD127^–^ from peripheral blood of healthy donors, transducing them retrovirally with an amphotropic version of MSCV retrovirus, containing the hCD40-AIR. During expansion via α-CD3/CD28 stimulation (TransAct) and IL-2, approximately 70% of human Treg cells expressed hCD40-AIR on their surface (Figures 6B and 6C), and FOXP3 expression remained stable throughout the process (Figure S5A). In co-culture experiments with human CD40L-expressing HEK cells, only the hCD40-AIR Treg cells showed activation as demonstrated by the induction of CD137 (4-1BB) and glycoprotein A repetitions predominant (GARP) expression (Figures 6D and 6E). The CD40L-dependent activation was absent in the non-transduced CD90.1^–^ fraction of Treg cells of the same co-cultures (Figures S5B and S5C), as well as in Treg cells expressing an irrelevant CAR (α-carcinoembryonic antigen) after co-culture with CD40L-expressing HEK cells (Figures 6D and 6E). Furthermore, stimulating hCD40-AIR Treg cells with CD40L upregulated LAP, CD69, and LAG3 to levels comparable with those seen after α-CD3/CD28 activation (Figure 6F). Finally, we performed suppression assays using hCD40-AIR Treg cells and control CEA CAR Treg cells and could observe that, in two of three donors, hCD40-AIR Treg cells were more efficient in suppressing Tconv proliferation in an antigen-independent system (Figure S5D).

These results demonstrate that human Treg cells expressing the hCD40-AIR sensor can effectively detect and respond to CD40L, similarly to their murine Treg counterparts. This illustrates the feasibility of CD40-AIR Treg therapy as a potential strategy for treating T cell-driven inflammatory diseases in humans.

## Discussion

Treg cells are key players in inducing and maintaining peripheral tolerance, thereby preventing excessive and harmful immune responses. Preclinical and clinical studies have already shown that adoptive transfer of Treg cells is safe, feasible, and can be an effective strategy for the treatment of autoimmune and inflammatory diseases, including transplant rejection.^11^ One of the modes of action of Treg therapy is the ability of Treg cells to induce cross-tolerance and infectious tolerance,^70^^,^^71^ a finding that was first introduced in the context of transplantation and has recently been confirmed in CAR Treg cells.^72^ Moreover, preclinical studies have highlighted that the efficacy of adoptive Treg cell therapy is enhanced when utilizing antigen-specific Treg cells, such as those engineered with CARs or recombinant TCRs that recognize disease-relevant antigens.^8^ These strategies are of particular interest in settings of solid organ transplantation, where graft-defined HLA antigens can be selected as targets. CAR Treg cell therapy targeting HLA-A2 is promising and is currently being tested in phase I clinical trials in patients who have undergone liver or kidney transplantation (NCT04817774, NCT05234190). In most inflammatory diseases, the disease-relevant antigens that could be targeted and are sufficiently and selectively expressed in affected organs are generally unknown. As a result, generating antigen-specific TCR- or CAR Treg cells for these conditions is a major challenge. To overcome this, we propose targeting CD40L expressed by activated T cells with CD40-AIR Treg cells. Self- or allo-reactive T cells, which are key drivers of disease, have been shown to express CD40L.^50^^,^^52^^,^^73^ In addition, under inflammatory conditions, other immune cell populations (e.g., platelets, NK cells, ILCs, B cells, monocytic cells, or mast cells),^74^^,^^75^^,^^76^^,^^77^^,^^78^ as well as innate immune-like cells (e.g., endothelial or epithelial cells),^38^ have been shown to upregulate CD40L expression. Moreover, CD40L-mediated activation of CD40-AIR Treg cells presumably happens in both lymphatic organs and in inflamed target tissues, enabling Treg cells to interfere on both levels during the pathogenic immune response. The exact mechanism of how CD40-AIR Treg cells can suppress CD40L-expressing effector T cells is not completely understood due to the complexity of Treg cell-mediated suppression. Various signals such as antigen, co-stimulation, and cytokines limit T cell activation and division. The impact of Treg cells is in eliminating or mitigating certain positive signals and in providing negative signals. Examples of reducing positive signals include CTLA4 binding to CD80/86 and IL-2 inhibition through absorption or decreased production. Treg cells also reduce CD80/86 expression directly on APCs to regulate co-stimulation strength. Examples of the addition of negative signals include TGF-β produced by Treg cells that act on effector T cells.^79^ As we clearly show that AIR activation induces LAP (TGFb1) expression as well as CTLA4 expression on RNA and protein level, these proteins and their pathways should play a role in CD40-AIR Treg-mediated suppression *in vivo*. In addition to these mechanisms of Treg-mediated suppression, a recent paper has suggested that CAR Treg cells can inhibit unwanted immune cell activation by removing ligands and parts of the cell membrane from target cells in a process called trogocytosis.^43^ Indeed, our *in vitro* data suggest that trogocytosis of CD40L by CD40-AIR Treg cells may interfere with APC effector T cell activation. Our data show reduced activity of donor CD4 and CD8 T cells isolated during the early phase of the GvHD located in the spleen, a lymphatic organ where allo-specific T cell priming and reactivation takes place. We observed a stronger suppression of donor T cell proliferation by the CD40-AIR compared with control Treg cells. Both Treg cell treatment groups had an influence on expression of certain effector, migration, and adhesion molecules in donor Tconv. While the role of CD40-AIR Treg cells have not been addressed in the graft-vs-leukemia response, our data showed that the severity and development of GvHD was strongly reduced or inhibited by CD40-AIR Treg cells, validating the therapeutic concept *in vivo*.

Although CD40-AIR and CAR Treg cells use different binding mechanisms, our results indicate that CD40-AIR signaling mirrors CAR Treg signaling and leads to full Treg cell activation. However, CD40-AIR therapy could offer several advantages: first, incorporation of a natural receptor domain potentially exhibits greater stability compared with CARs, which are prone to aggregation via their single-chain variable fragments, potentially triggering unwanted tonic signals.^80^ Second, the CD40-AIR becomes activated only by membrane-bound CD40L and not by its soluble ligand. This is of importance for CD40-AIR Treg activity since it is locally restricted to areas with activated T cells, such as GvHD, where alloreactive donor T cells are present. Activation occurs both in lymphoid organs during T cell priming and at inflamed tissue sites, offering a two-tiered response that CAR- or TCR-Treg cells may lack, as they primarily target antigens expressed in specific tissues. Third, CD40-AIR signaling is transient and timely restricted signaling, occurring only when its ligand, CD40L, is present. This avoids the continuous activation and potential exhaustion seen with tonic CAR signaling.^81^ Fourth, the overall CD40-AIR Treg cell activity is linked to the actual inflammation level, as more activated T cells offer more CD40L, which in turn can better activate the Treg cells. In contrast, CAR Treg cells are activated by antigen expression, regardless of inflammation level. Nevertheless, both CAR- and AIR Treg strategies have their merits and may complement each other in certain diseases. CAR Treg cells may be more suited for solid organ transplantation, where a well-defined antigen exists, whereas CD40-AIR Treg cells may be more effective in T cell-mediated inflammatory diseases, where antigen targets are unknown, and inflammation affects multiple organs episodically.

Recent strategies to treat antibody-mediated autoimmune diseases, such as using α-CD19-CAR T cells to eliminate pathogenic CD19^+^ B cells have been applied in patients.^82^ First clinical data look promising for the treatment of lupus erythematosus, idiopathic inflammatory myositis, systemic sclerosis, and myasthenia gravis. However, this cell-killing approach would be inappropriate for T cell-mediated autoimmune diseases, as loss of CD4^+^ T cells, as seen in untreated HIV, can lead to severe infections and tumor development.^83^ Instead, CD40-AIR Treg cells sense activated T cells and convert this signal into Treg activation, curbing inflammation without removing essential T cells. The CD40L-CD40 interaction is an ideal target for this purpose.

Given the critical importance of CD40L-CD40 signaling in several autoimmune, chronic inflammatory, neurological, and neurodegenerative disease indications led us to select CD40L as an AIR target.^13^^,^^84^^,^^85^ As the CD40 pathway is an attractive therapeutic target, several clinical trials in which either the ligand or receptor is blocked by monoclonal antibodies have been initiated.^14^^,^^29^ For example, pharmaceutical companies are currently testing CD40L blocking antibodies in a phase II/III clinical trial in MS and SLE patients (MS: NCT04879628 NCT06141473, NCT06141486; SLE: NCT05039840 NCT04976322, NCT04294667) and phase I clinical trials for the prevention of GvHD (NCT03605927) and the treatment of amyotrophic lateral sclerosis (NCT04322149) were recently completed.

Genetically modified CAR- or TCR-Treg cells as cell therapeutics are gaining enormous interest and both biotech and pharmaceutical companies and companies have made remarkable investments in this field.^86^ In addition to CAR- and TCR-Treg engineering, we propose here the synthetic immune receptor CD40-AIR as a sensor for Treg cells, thus introducing an alternative treatment strategy for various T cell-mediated inflammatory and autoimmune diseases.

## Materials and methods

A list of critical commercial assays can be found in Table S6. A list of chemicals, plasmids, and recombinant proteins used can be found in Table S7. A list of software and algorithms used can be found in Table S8.

### Ethics statement

Peripheral blood mononuclear cells from blood donors were isolated from leukocyte reduction chambers from healthy donors donating thrombocytes. Collection of immune cells from those donors was performed in compliance with the Helsinki Declaration after ethical approval by the local ethical committee (Regensburg University, reference number 13-0240-101 and 19-1414-101) and signed informed consent.

### Peripheral blood mononuclear cell isolation and enrichment of blood lymphocytes

T cells were isolated from human blood, leukocyte reduction chambers. At first, leukocytes were diluted 1:1 with PBS and the mixture was split into four fractions and underlaid with an equal amount of Pancoll (PAN Biotech). The samples were centrifuged at 1,000 × *g* for 20 min at RT (without brake). Then, the PBMC layer was isolated and washed twice by centrifugation steps. Cells were pre-enriched with a-human CD25-PE (clone 2A3) followed by column-based magnetic separation using a-PE ultrapure microbeads (Miltenyi Biotec) following the manufacturer’s protocol.

### Mice

Female BALB/c mice, C57BL/6 Nr4a1-eGFP mice (JAX stock no. 016617, C57BL/6-Tg(Nr4a1-EGFP/cre)820Khog/J) and C57BL/6 CD45.1^+^ mice (JAX stock no. 002014, B6.SJL-PtprcaPepcb/BoyCrl) were obtained from Charles River Breeding Laboratories (Wilmington, MA) or the Jackson Laboratory (Bar Harbor, ME). B6N.129(Cg)-Foxp3tm3Ayr mice (Foxp3.IRES-DTR/GFP) were bred to C57BL/6 CD45.1^+^ mice and served as BM donors for GvHD experiments. C57BL/6 Foxp3-hCD2 (Foxp3tm1(CD2/CD52)Shori) were a gift from S. Hori.^87^

Animals were housed under specific pathogen-free conditions at the Regensburg University Clinics animal care facility, and the governmental committee for animal experimentation (Regierungspräsidium Unterfranken) approved all experiments involving animals.

### Design of CD40 AIR

Extracellular and transmembrane domains of CD40 were fused to the intracellular signaling domains of CD28, CD137, or ICOS and the CD3-ζ-chain. Two point mutations were introduced into the murine CD28 domain as they were shown to increase expression of CARs.^88^ For the generation of human and murine AIRs, nucleotide sequences published on https://www.ensembl.org were used. The α-HLA-A2 CAR construct is based on published sequence (GenBank: MP143507.1). The α-CD19 CAR as well as the α-CEA CAR have been described previously.^89^^,^^90^ ORFs coding for the a-CD19 CAR, as well as the AIRs and the a-HLA A2 CAR and the a-CEA CAR were fused to the congenic marker CD90.1 by ligating to a self-cleaving P2A sequence. cDNAs were synthesized by Thermo Fisher/Life Technologies and cloned into the pMSCV-Thy1.1 retroviral backbone (Addgene, catalog no. 17442) via NotI/Mlu.

### Preparation of samples for flow cytometry

Preparation of single-cell solutions and pre-enrichment as described previously.^12^ Samples were stained either in 1.5 mL Eppendorf tubes or 96-well plates in FACS buffer (1% FCS in PBS). Surface staining was performed at 4°C for 20 min in 50–100 μL staining volume. The following antibodies were used for surface staining of murine samples: TCR-β-chain (H57-597), CD4 (RM4-5), CD8α (53-6.7), CD19 (6D5), CD25 (PC61), CD45.1 (A20), CD45.2 (104), CD90.1 (OX-7), KLRG1 (2F1), TIGIT (1G9), CD62L (MEL-14), CD137 (I7B5), CD69 (H1.2F3), LAP (TW7-16B4), H-2Kb (AF6-88.5), hCD2 (RPA-2.10), CD223 (C9B7W), CD154 (MR1), and CD40 (3/23).

The following antibodies were used for surface staining of human samples: CD4 (OKT4), CD25 (BC96, 2A3), CD127 (A019D5), CD45RO (UCHL1), CD45RA (HI100), CD137 (4B4-1), TCR-β-chain (IP26), LAG3 (11C3C65), GARP (7B11), LAP (FNLAP), CD69 (FN50), and CD40 (5C3).

Intracellular staining was performed with the Foxp3/Transcription Factor Buffer Set (eBiosciences) according to the manufacturer’s protocol with the following adaptations: intracellular staining was performed for 1 h at room temperature. Antibodies for intracellular staining include FOXP3 (JFK-16S) and CD152 (UC10-4B9) for mouse and FOXP3 (206D) for human cells. Dead cells were excluded with a fixable live/dead dye (eBioscience Fixable Viability Dye eFluor780).

### Flow cytometry and FACS sorting of T cells from blood, tissues, and cell cultures

Cells were pre-enriched and stained as described previously.^12^ Afterward, samples were filtered with a 40 μM filter unit and acquired on a BD FACSymphony or a BD FACSCelesta flow cytometer. Fluorescence spillover compensation was performed with lymphocytes stained with a-CD4 antibodies in the respective colors and BD CS&T beads were used to validate machine functionality. Flow cytometry data were analyzed using BD FlowJo (version 10.9.0). Sorting was performed with a BD FACSFusion cell sorter with a 70 μm nozzle. Post-sort quality controls were performed as applicable. For murine Treg cell expansion cultures, a-CD25 enriched cells were sorted for naive CD4^+^CD25^+^CD62L^+^ Treg cells or a-hCD2 pre-enriched cells were sorted for CD4^+^CD25^+^hCD2^+^. For cultivation, cells were sorted directly into cell culture medium.

For bulk RNA sequencing of AIR/CAR-activated Treg cells, 2–5 × 10^3^ cells were sorted on live CD4^+^CD25^+^CD90.1^+^GFP(Nr4a1)^+^CD137^+^ or CD4^+^CD25^+^CD90.1^+^ directly into 350 μL RLT+ lysis buffer (QIAGEN RNEasy Plus Micro Kit no. 74034). For bulk RNA sequencing of re-isolated T cells, 1 × 10^4^ live CD45.1^+^ hCD2^–^ CD4^+^ cells from spleens, isolated from GvHD animals 7 days after BMT, were sorted into 350 μL RLT+ lysis buffer. For human Treg cultures, a-CD25 pre-enriched cells from human blood were sorted on live TCR-β^+^ CD4^+^ CD25^+^ CD127^–^ directly into TexMACS medium.

### Murine and human Treg cell cultures

For murine Treg cultures, sorted Treg cells were seeded at 3 × 10^4^ to 4 × 10^4^ cells per well in a 96-well round-bottom plate with α-CD3/CD28 beads (Miltenyi Biotec, 4 beads per cell) and 2,000 U/mL rhIL-2 (Proleukin S, Novartis). DMEM (Gibco/Invitrogen) was used as culture medium supplemented with 10% FCS, 2 mM L-glutamine, 5 × 10^−5^ M 2-ME, MEM-Vitamin Sol., 10 mM HEPES, 100 mM sodium-pyruvate, 1% NEAA (PAN Biotech), 100 U/mL penicillin-streptomycin (Gibco). Murine Treg cells were transduced 48 h after isolation. For restimulation assays α-CD3/CD28 beads were removed from Treg cell cultures via a MACSiMAG separator magnet (Miltenyi Biotec), washed with medium, and afterward seeded without TCR stimulation in fresh medium supplemented with rhIL-2 (100 U/mL) and rested for 24 h.

Human sorted Treg cells were cultured in TexMACS medium (Miltenyi Biotec) supplemented with 500 U/mL rhIL-2 (Proleukin S, Novartis) and TransAct (1:100 diluted). Human Treg cells were transduced 48 h after isolation. For restimulation assays Treg cells were washed twice with TexMACS medium and afterward seeded in fresh medium supplemented with rhIL-2 (100 U/mL) and rested for 24 h.

### Digestion of tissues for flow cytometric analysis and FACS sorting of cells

To harvest and prepare singe-cell solutions from intestine, colon tissue of transplanted Balb/c mice was removed and cleared of feces. Cells were isolated according to the manufacturer’s instructions with the lamina propria dissociation kit (Miltenyi Biotec) and gentleMACS device (Miltenyi Biotec, program 37C_mLPDK_1). More detailed protocols about T cell isolation from tissues have been published.^91^

### Retroviral transduction of Treg cells

Phoenix-Eco cells were seeded on a gelatin matrix at 1.3 × 10^6^ cells per well in a 6-well plate 6 h before lipofection. To produce liposomal particles containing the viral transgene, 3 μg of vector DNA and 1 μg of additional pCL-Eco packaging plasmid were coincubated with 12 μL of TransIT-293 transfection reagent (MoBiTec) for 20 min in OptiMEM medium at RT. Liposomes were added to Phoenix-Eco cells and incubated for an additional 16 h. Afterward medium was exchanged and production of viral particles could proceed for 24 h. Then supernatant with produced pMSCV retrovirus was added to Treg cell cultures and mixed gently. Treg cells were transduced for 6.5 h incubation at 37°C. Afterward, viral supernatant was removed and cells were incubated with fresh medium supplemented with IL-2 (2,000 U/mL) for another 72–96 h. Then, cells were harvested and a-CD3/CD28 antibody-coupled beads were removed by using a MACSiMAG separator magnet (Miltenyi Biotec). For transduction of human Treg cells an amphotrophic Phoenix cell line was used. Phoenix-Ampho cells were seeded at 1.3 × 10^6^ cells per well in a 6-well plate 6 h before lipofection. For production of liposomal particles containing the viral transgene, 3 μg of vector DNA and 1 μg of additional pCL-Ampho packaging plasmid were coincubated with 12 μL of TransIT-293 transfection reagent (MoBiTec) for 20 min in OptiMEM medium at RT. Liposomes were added to Phoenix-Ampho cells and incubated for an additional 16 h. Afterward, medium of was exchanged and production of viral particles could proceed for 24 h. Then supernatant with produced pMSCV retrovirus was added to Treg cell cultures and mixed gently. Treg cells were transduced for 6.5 h incubation at 37°C. Afterward, viral supernatant was removed and cells were incubated with fresh medium supplemented with IL-2 (500 U/mL), 100 U/mL penicillin-streptomycin, and fresh TransAct (1:100 diluted) for another 72–96 h.

### Transient transfection of HEK293 cells with CD154 (CD40L)

For production of liposomal particles, 2 μg of plasmid DNA was incubated with 6 μL TransIT-293 transfection reagent in 100 μL OptiMEM medium for 20 min at room temperature. Then liposomes were gently added to 5 × 10^5^ HEK293 cells resuspended in 500 μL DMEM. After shaking for 1 min, 230 μL DMEM was added and 100 μL of the cell-liposome suspension (containing approximately 6 × 10^4^ cells) was added per well into a 96-well flat-bottom plate. Eighteen hours after lipofection, medium was changed and transfected HEK293 cells could be used for co-culture experiments.

### CD40 AIR-mediated trogocytosis assay

CD40L-expressing HEK293 cells were seeded at a concentration of 60,000 cells/well in a flat-bottom 96-well plate, precoated with 0.1% gelatin for 30 min at RT. Eighteen hours after initial seeding, supernatants were carefully removed and cells were stained with anti-mouse CD154 (CD40L; clone MR1) antibody conjugated to APC at a concentration of 1:100 for 20 min at 37°C, 5% CO_2_. Cells were gently washed 3× with warm T cell medium for 10 min at 37°C, 5% CO_2_. Subsequently, 100,000 CD40 AIR or truncated AIR Treg cells were added to each well. Cells were centrifuged briefly (300 × *g*, 2 min) to encourage cell-cell interactions and co-cultured at 37°C, 5% CO_2_. After 3 h, cells were harvested and stained for flow cytometric analysis as described above.

### Proliferation assay

The Miltenyi MACSiMAG separator magnet was used to remove α-CD3/CD28 antibody-coupled beads from Treg cell cultures. Treg cells were rested for 18 h in fresh medium supplemented with 100 U/mL rhIL-2. Treg cells were labeled with CellTrace CFSE Cell Proliferation dye (1 μM) and added onto HEK293 wild-type cells or cells expressing murine CD40L. rhIL-2 (2,000 U/mL) was added to the cell cultures. After 72 h of coincubation, proliferation of transduced, CD90.1^+^ CFSE-labeled Treg cells was analyzed via flow cytometry.

### Suppression assay

Human Tconv cells were isolated by CD4-positive enrichment and labeled with CellTrace CFSE Cell Proliferation dye (1 μM). Tconv cells were stimulated for 6 h with TransAct. Stimulation was removed by washing and cells were co-cultured with either CD40-AIR Treg cells or CEA CAR Treg cells at a ratio of 1:2 Treg:Tconv for 12 h. As a positive control Tconv cells without Treg cells were cultured. Proliferation of Tconv cells was evaluated after 7 days by flow cytometry.

### GvHD model

Female BALB/c (H-2K^d^) recipients were myelobalatively irradiated with 8 Gy and intravenously injected with 2.5 × 10^6^ BM cells only (BM control) or together with 5 × 10^5^ splenocytes from C57BL/6 CD45.1^+^ donors (H-2K^b^). The animals in the therapy groups received 2.5 × 10^5^ *in-vitro*-expanded and transduced C57BL/6 Treg (Foxp3-hCD2 reporter, CD45.2^+^). Recipients were monitored daily, and body weight and GvHD symptoms assessed two or three times weekly by nonblinded investigators applying a standardized scoring protocol which was approved by Committee on Ethics of Animal Experiments at the Bavarian Government. GvHD score was defined as follows: loss of body weight >10% = 15, >20% = 30, > 25% = 40; activity reduced = 5, strongly reduced = 10, activity only after stimulation = 15, no activity after stimulation = 40; fur ruffled ventral = 5, fur ruffled ventral and dorsal = 10, fur ruffled over 50% = 15, fur completely ruffled = 20; kyphosis in resting phase = 5, in movement = 10, during standing on hind legs = 15, very strong, no mobility = 20. Animals possessing a summarized score of 40 had to be euthanized.

### Cell lines

Phoenix-Eco and Phoenix-Ampho cell lines were purchased from ATCC (catalog nos. CRL-3214 and CRL-3213). These are second-generation retrovirus producer cell lines for the generation of ecotropic and amphotropic retroviruses.

### RNA-seq and bioinformatics

Total RNA was isolated using the QIAGEN Rneasy Micro Kit, and RNA was eluted in 14 μL RNase-free water. RNA quality was assessed using the Tapestation system 4200 and High-Sensitivity RNA screentape (Agilent). Seven microliters of the RNA was used for generating RNA-seq libraries using the SMART-seq Stranded Kit from Takara. Indexed libraries were pooled in an equimolar ratio and sequenced on an Illumina NextSeq 550 machine with NextSeq 500/550 High Output Kit v.2.5 (75 cycles). Quality control and read mapping to the mouse reference genome (GRCm38, gencode, release23) was performed using the SnakePipes analysis pipeline (v.2.5.1). Mapping was performed using STAR, and gene counts are based on featureCounts. Analyis was performed in R (v.4.1.1)/Bioconductor. Quality control of the count matrix and differential expression gene calling was performed with DESeq2 (v.1.34.0). Gene counts were imported and prefiltered with edgeR:filterByExpr, and the false discovery rate was set to 0.05. Gene ontology analysis was performed with genes upregulated with a log2 fold change >0.5 and FDR <0.05 using enrichR (v.3.1). Gene set enrichment analysis was performed using fgsea with fdr 0.1. Plots were created with ggplot2 (v.3.3.5) and EnhancedVolcano (v.1.12.0).

### Statistical analysis

Data were analyzed with Prism software or algorithm. Statistical details are indicated in the figure legends. For survival differences, Kaplan-Meier analysis was performed and the log rank test was used. Statistics for RNA sequencing as described in bioinformatics methods. *p* values < 0.05 were considered significant (∗*p* < 0.05, ∗∗*p* < 0.01, ∗∗∗*p* < 0.001).

## Data and code availability

• Correspondence and requests for materials should be addressed to Markus Feuerer.
• The main data supporting the results in this study are available within the paper and its supplemental information.
• The accession numbers for the RNA-seq data reported in this paper are: Gene Expression Omnibus (GEO): GSE279659.

## Acknowledgments

This work was funded by the 10.13039/501100001659Deutsche Forschungsgemeinschaft (DFG, 10.13039/501100001659German Research Foundation) Projektnummer 324392634 - TRR 221 to M.F., P.H., M.E., M.R., and A.B. Sequencing and bioinformatic analysis were conducted at the NGS Core Unit of the Leibniz Institute for Immunotherapy (LIT). We thank the flow cytometry core facility of the LIT and the animal facility of the University of Regensburg for technical support. We thank H. Stanewsky, J. Raithel, U. Ackermann, K. Gütter, M. Wuttke, R. Eder, and I. Fink for technical support. We thank S. Hori for Foxp3-hCD2 mice.

## Author contributions

Conceptualization, S.B., T.H., and M.F.; methodology and investigation, S.B., B.R., V.H., P.H., N.S., I.H.-L., P.S., L.S., B.E., and F.H.; visualization, S.B., I.H.-L., N.S., and L.S.; funding acquisition, M.F.; supervision, S.B., T.H., and M.F.; writing – original draft, S.B. and M.F.; writing – review & editing, S.B., M.F., T.H., L.S., P.S., A.B., M.E., P.H., and M.R.

## Declaration of interests

S.B., T.H., and M.F. are inventors on patent application (patent application no. WO 2023/227521) based on technology presented in this manuscript.

## Supplemental information

Document S1. Figures S1–S5 and Tables S6–S8

Table S1. CD40 AIR-regulated genets

Table S2. HLA-A2 CAR-regulated genes

Table S3. DE genes in control Tconv vs. GvHD Tconv

Table S4. DE genes in CD40 Tconv vs. GvHD Tconv

Table S5. DE genes in CD40 Tconv vs. control Tconv

Document S2. Article plus supplemental information
