# Supplementary material for: Engineered CD40-biosensor-expressing Treg cells as a cell therapy approach for inflammatory diseases
Source: Mol Ther. 2026 Jan 10;34(4):2217–35. doi: 10.1016/j.ymthe.2026.01.002 (PMC13069339; doi:10.1016/j.ymthe.2026.01.002)
Supplement: Supplementary file 1 [file mmc1.pdf]

YMTHE, Volume *34*

Supplemental Information

Engineered CD40-biosensor-expressing Treg cells as a cell therapy approach for inflammatory diseases

Sebastian Bittner, Lisa Schmidleithner, Brigitte Ruhland, Veronika Hofmann, Philipp Stüve, Frauke Hoffmann, Bernd Echtenacher, Petra Hoffmann, Matthias Edinger, Andreas Beilhack, Nicholas Strieder, Inmaculada Hernandez-Lopez, Michael Rehli, Thomas Hehlgans, and Markus Feuerer

A

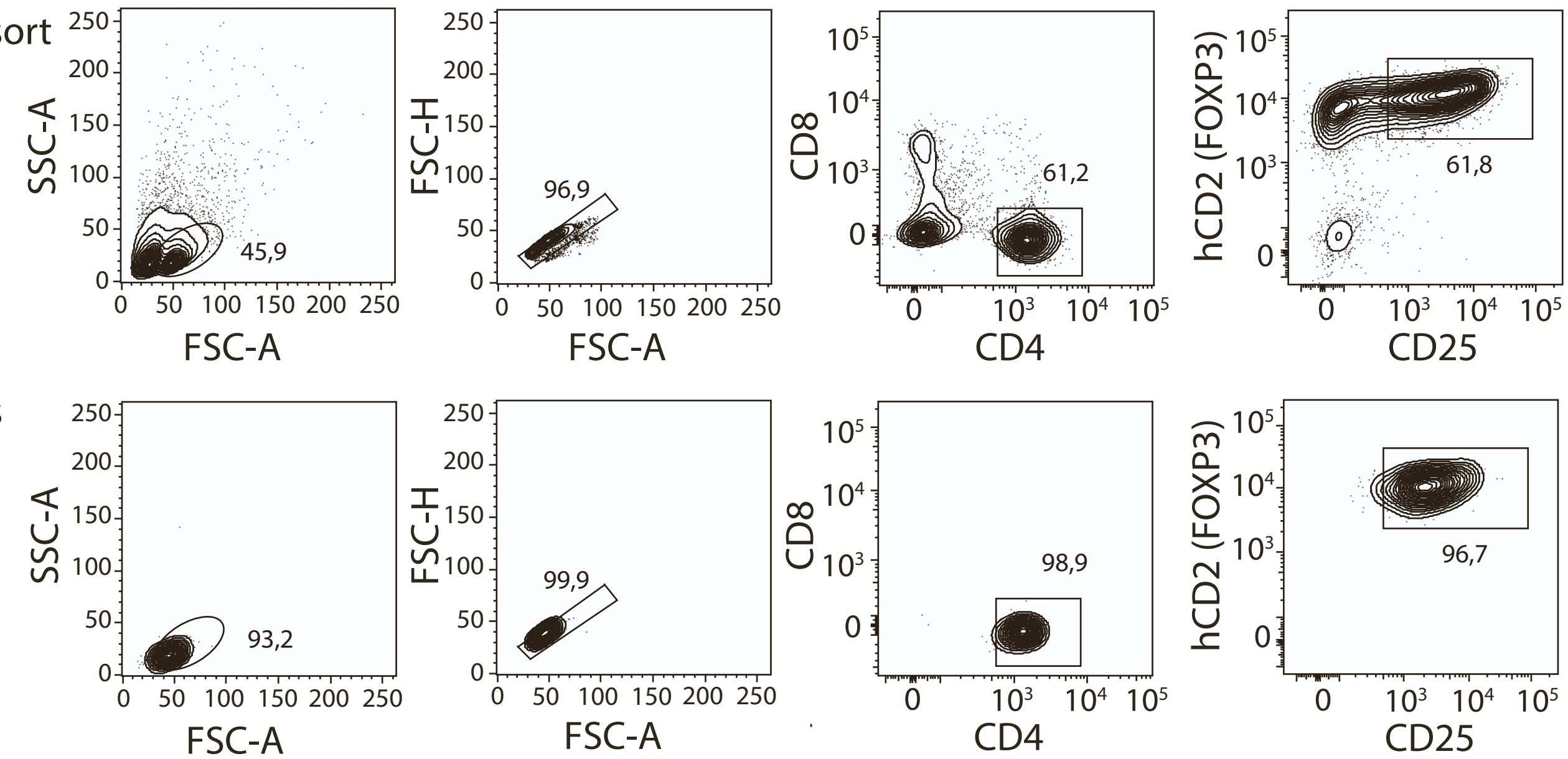

Treg cell sort
reanalysis
SSC-A
FSC-A
FSC-H
CD8
CD4
hCD2 (FOXP3)
CD25
45,9
96,9
61,2
61,8
93,2
99,9
98,9
96,7

B

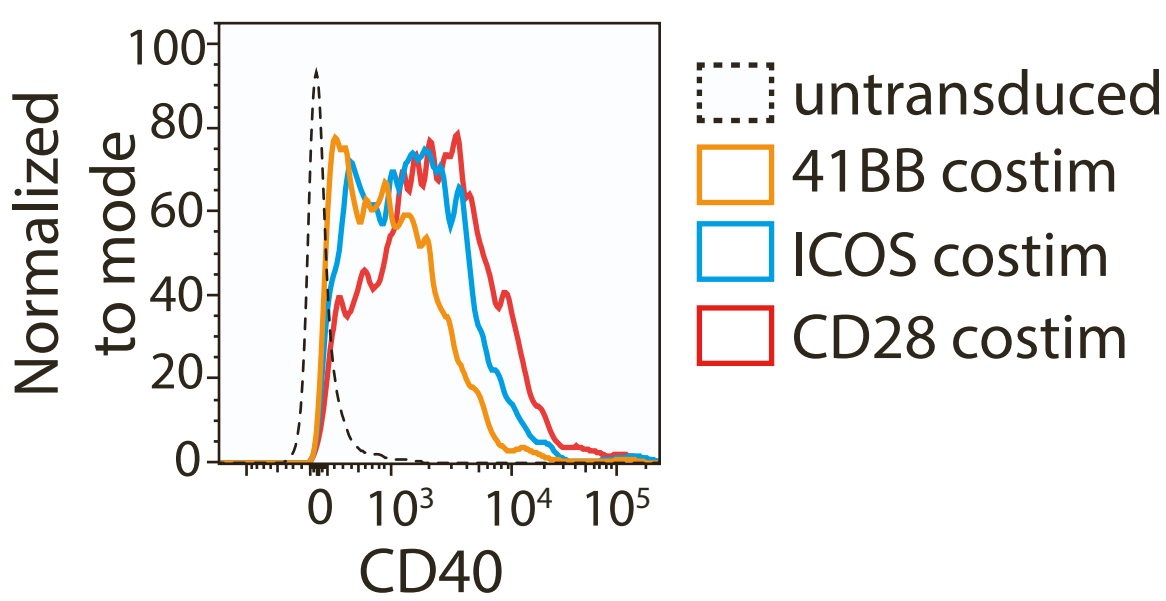

Normalized to mode
CD40
untransduced
41BB costim
ICOS costim
CD28 costim

Figure S1. FACS-sorting of Treg cells for cultivation

(A) Representative flow cytometry data showing the sorting strategy for Treg cells from single cell solution prepared from spleen and lymph nodes of FOXP3-hCD2 reporter mice. $hCD2^+$ pre-enriched cells were sorted on $CD4^+CD25^+hCD2^+$ to high purity (top panel). Re-analysis was performed to control purity of sorted Treg cells (lower panel). (B) Representative flow cytometric analysis of murine Treg cell cultures. CD40-AIR surface expression in transduced $CD90.1^+$ Treg cells 3 days after transduction with CD28, ICOS or 41BB containing AIR construct is shown. (C) Analysis of CD137 and TIGIT expression of the cell fraction expressing intermediate levels of AIR (derived from Fig. 1 F-H). Gating (above) and quantification of CD137 and TIGIT expression (below) (n=3; unpaired t test).

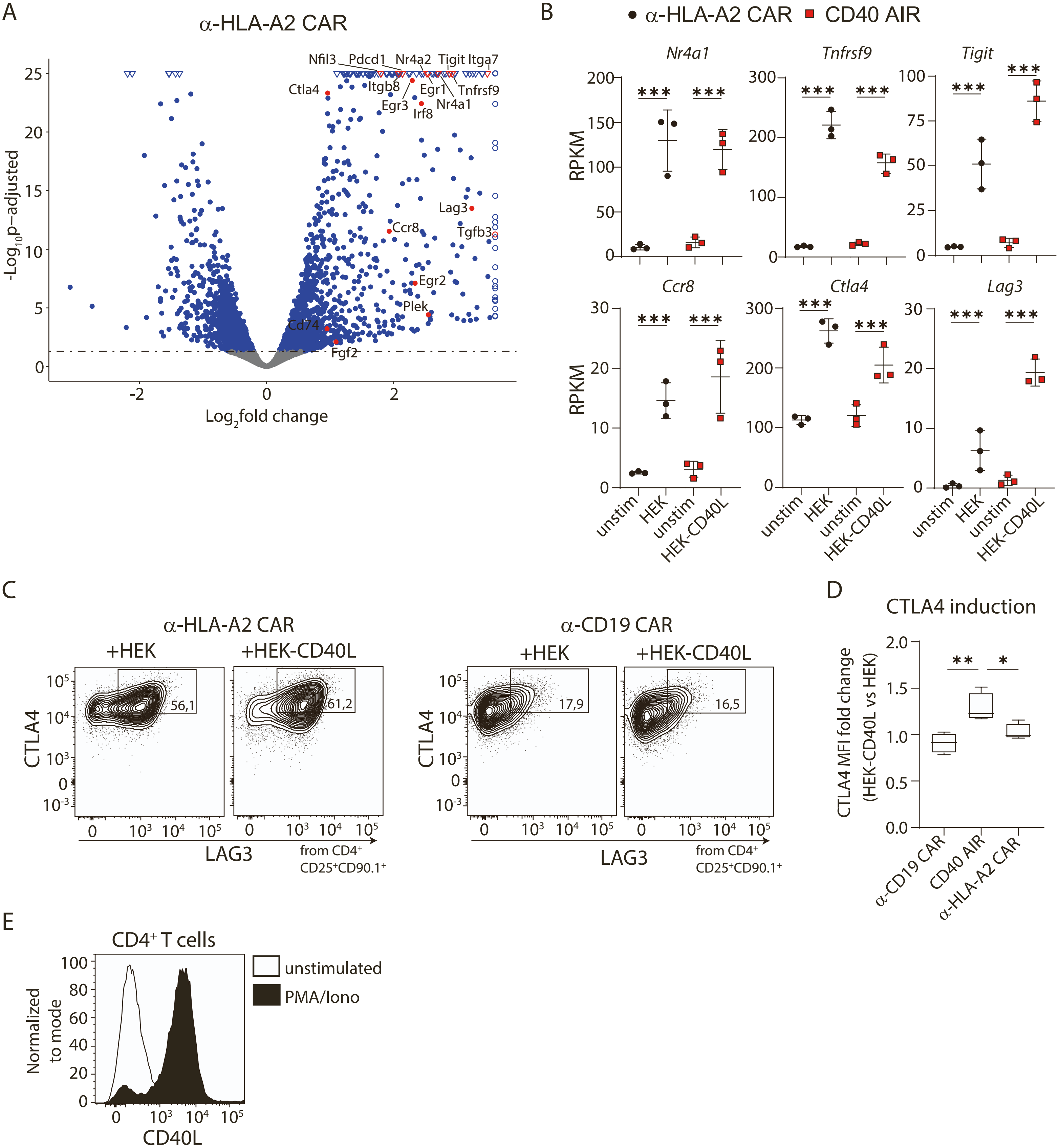
A
α-HLA-A2 CAR
Nfil3
Pdcd1
Nr4a2
Tigit
Itga7
Ctla4
Itgb8
Egr1
Tnfrsf9
Egr3
Nr4a1
Irf8
Lag3
Ccr8
Tgfb3
Egr2
Plek
Cd74
Fgf2
-Log10p-adjusted
Log2fold change
B
α-HLA-A2 CAR
CD40 AIR
Nr4a1
Tnfrsf9
Tigit
Ccr8
Ctla4
Lag3
RPKM
unstim
HEK
unstim
HEK-CD40L
C
α-HLA-A2 CAR
+HEK
+HEK-CD40L
56,1
61,2
α-CD19 CAR
17,9
16,5
CTLA4
LAG3
from CD4+ CD25+CD90.1+
D
CTLA4 induction
CTLA4 MFI fold change (HEK-CD40L vs HEK)
α-CD19 CAR
CD40 AIR
α-HLA-A2 CAR
E
CD4+ T cells
unstimulated
PMA/Iono
Normalized to mode
CD40L

Figure S2. Characterization of CAR and AIR signaling in Treg cells

(A) Volcano plot showing differentially expressed transcripts after α-HLA-A2 CAR stimulation. α-HLA A2 CAR-expressing Treg cells were rested for 24 h and then cocultured for 18 h with HEK cells or left unstimulated. Treg cells were sorted on living $CD4^+CD25^+CD90.1^+$ (unstimulated) or $CD4^+CD25^+CD90.1^+Nr4a1^+CD137^+$ (CAR activated, complete list Table S 2). (B) RNA expression data for *Nr4a1*, *Tnfrsf9*, *Tigit*, *Ccr8*, *Ctla4* and *Lag3* from unstimulated and stimulated (described in A) CD40-AIR or α-HLA A2 CAR Treg cells (complete list Table S1 and S2, Deseq2). (C) Flow cytometric analysis from α-HLA-A2 CAR and α-CD19 CAR-expressing Treg cells after 18 h coculture with HEK ± CD40L. Protein expression of LAG3 and intracellular CTLA4 is shown. (D) Fold increase in CTLA4 MFI was calculated in CD40-AIR, a-HLA-A2 CAR and a-CD19 CAR Treg cells after 18 h coculture with HEK ± CD40L (CD40-AIR n=3, α-HLA-A2 CAR n=3, α-CD19 CAR n=3, one-way ANOVA). (E) Flow cytometric analysis of primary $CD4^+$ T cells ± PMA/Ionomycin (eBiosciences Cell stimulation cocktail plus Transport Inhibitor) stimulation for 3 h. Intracellular CD40L expression is shown. (F) CD40L expressing HEK293T cells were stained for CD40L before co-culture with truncated or CD40 AIR cells. 3 hours after co-culture, transfer of CD40L protein to AIR cells was quantified by flow cytometry. Left, exemplary FACS plot, right, quantification. Paired student's t test. Data are derived from 3 biological replicates and 2 individual experiments

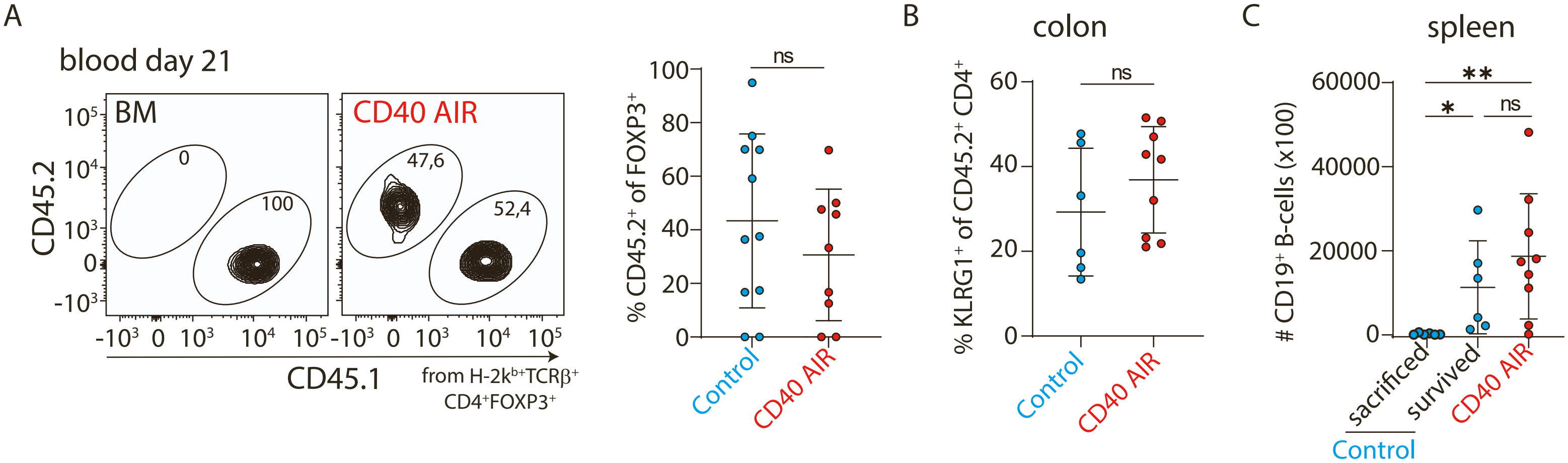
A
blood day 21
BM
CD40 AIR
0
100
47,6
52,4
CD45.2
CD45.1
from H-2k^b+TCRβ+ CD4+FOXP3+
% CD45.2+ of FOXP3+
ns
Control
CD40 AIR
B
colon
% KLRG1+ of CD45.2+ CD4+
ns
Control
CD40 AIR
C
spleen
CD19+ B-cells (x100)
**
*
ns
sacrificed
survived
CD40 AIR
Control

Figure S3. CD40-AIR Treg cells ameliorate GvHD pathology in mice

(A) On day 21, blood samples from transplanted animals were taken to check for transferred CD45.2$^+$ cells within the reconstituting FOXP3$^+$ Treg compartment via flow cytometry. Left: Representative flow cytometric data of blood samples form mice with and without Treg transfer. Right: Quantification of transferred Treg cells in blood samples, graph contains datasets from two independent experiments (Mann Whitney U test, n = 9–11). (B) Isolated cells from digested colons of CD40-AIR or control Treg cell treated mice were analyzed via flow cytometry at day 47. Frequency of KLRG1$^+$ transferred CD45.2 Treg cells was analyzed (control n=6, CD40-AIR n=9; Mann-Whitney U). (C) Single cells suspensions from spleens of mice receiving CD40-AIR or control Treg cell treatment were analyzed via flow cytometry at time point of scarification or at the end of the experiment (day 47). (Kruskal-Wallis test, control sacrificed n=6, control survived n=6, CD40-AIR n=9).

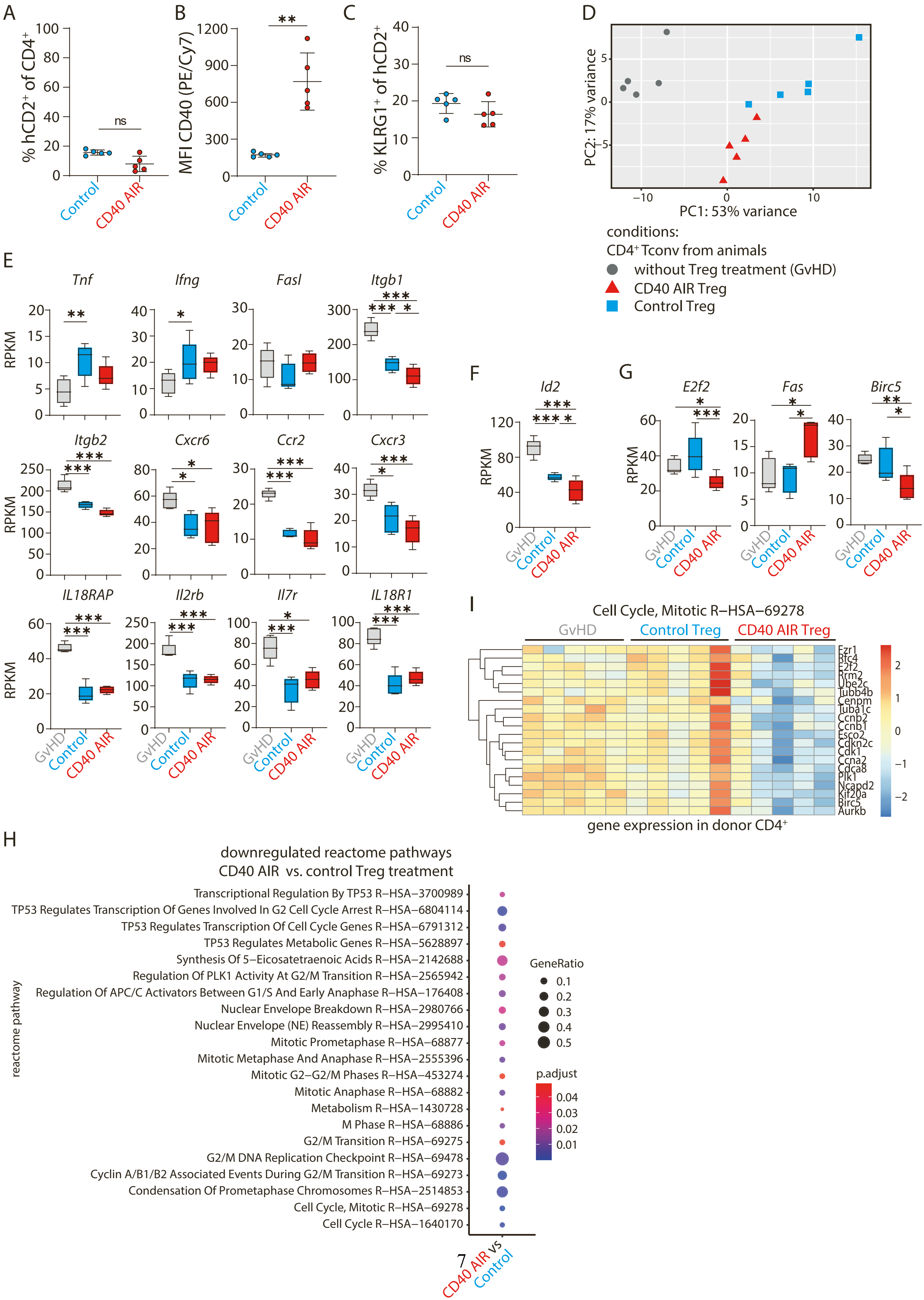

A
% hCD2+ of CD4+
ns
Control
CD40 AIR
B
MFI CD40 (PE/Cy7)
**
C
% KLRG1+ of hCD2+
ns
D
PC2: 17% variance
PC1: 53% variance
conditions:
CD4+ Tconv from animals
without Treg treatment (GvHD)
CD40 AIR Treg
Control Treg
E
Tnf
Ifng
Fasl
Itgb1
Itgb2
Cxcr6
Ccr2
Cxcr3
IL18RAP
Il2rb
Il7r
IL18R1
RPKM
GvHD
F
Id2
G
E2f2
Fas
Birc5
I
Cell Cycle, Mitotic R–HSA–69278
GvHD
Control Treg
CD40 AIR Treg
Ezr1
Rfc4
E2f2
Rrm2
Ube2c
Tubb4b
Cenpm
Tuba1c
Ccnb2
Ccnb1
Esco2
Cdkn2c
Cdk1
Ccna2
Cdca8
Plk1
Ncapd2
Kif20a
Birc5
Aurkb
gene expression in donor CD4+
H
downregulated reactome pathways
CD40 AIR vs. control Treg treatment
reactome pathway
Transcriptional Regulation By TP53 R–HSA–3700989
TP53 Regulates Transcription Of Genes Involved In G2 Cell Cycle Arrest R–HSA–6804114
TP53 Regulates Transcription Of Cell Cycle Genes R–HSA–6791312
TP53 Regulates Metabolic Genes R–HSA–5628897
Synthesis Of 5–Eicosatetraenoic Acids R–HSA–2142688
Regulation Of PLK1 Activity At G2/M Transition R–HSA–2565942
Regulation Of APC/C Activators Between G1/S And Early Anaphase R–HSA–176408
Nuclear Envelope Breakdown R–HSA–2980766
Nuclear Envelope (NE) Reassembly R–HSA–2995410
Mitotic Prometaphase R–HSA–68877
Mitotic Metaphase And Anaphase R–HSA–2555396
Mitotic G2–G2/M Phases R–HSA–453274
Mitotic Anaphase R–HSA–68882
Metabolism R–HSA–1430728
M Phase R–HSA–68886
G2/M Transition R–HSA–69275
G2/M DNA Replication Checkpoint R–HSA–69478
Cyclin A/B1/B2 Associated Events During G2/M Transition R–HSA–69273
Condensation Of Prometaphase Chromosomes R–HSA–2514853
Cell Cycle, Mitotic R–HSA–69278
Cell Cycle R–HSA–1640170
GeneRatio
p.adjust
7
CD40 AIR vs Control

Figure S4. CD40-AIR Treg cells suppress cell cycle and function of donor CD4^{+} T cell

(A) Summarized data showing frequencies of transferred hCD2^{+} Treg cells among CD4^{+} T cells in spleens of CD40-AIR or control Treg cell receiving mice 7 days after transplantation (Control n=5, CD40-AIR n=5; Mann-Whitney U). (B) Transferred hCD2^{+}CD90.1^{+} Treg cells were analyzed with flow cytometry for CD40 expression. Summarized data for mean fluorescence intensity of CD40 staining with PE/Cy7-conjugated antibodies are shown (Control n=5, CD40-AIR n=5; Mann-Whitney U). (C) KLRG1 expression in transferred hCD2^{+} Treg cells was analyzed with flow cytometry. Summarized data for frequencies of KLRG1 expression are shown (control n=5, CD40-AIR n=5; Mann-Whitney U). (D) 7 days after transplantation, donor-derived (H-2k^{b+}CD45.1^{+}) TCR^{+}hCD2^{-}CD4^{+} T cells were FACS sorted and RNA-sequencing was performed. Principal-component analysis of RNA-seq data of sorted CD4^{+} T cells from mice receiving no Treg cell treatment (GvHD), control Treg cell or CD40-AIR Treg cell treatment. RNA expression data for single genes from T cells isolated from mice receiving no treatment, Control Treg cells or CD40-AIR Treg cells are shown in (E), (F) and (G) (GvHD n=5, control n=5, CD40-AIR n=5; Deseq2). (H) Direct comparison of downregulated pathways in CD4^{+} T cells from CD40-AIR compared to control Treg treated mice. Significant enrichR comparison terms from GO Biological Process 2021 database are plotted (adjusted pvalue < 0.05, manually selected pathways based on biological relevance are shown, complete list is show in Table S 5). (I) VST-transformed scaled expression values of genes from the GO term mitotic cell cycle phase transition (GO:0044772), identified in (H), were plotted individually in a heatmap. Only genes significantly downregulated (adjusted pvalue < 0.05) in CD40-AIR Treg treated mice (comparison to control Treg treated mice) and not significant (adjusted pvalue > 0.05) in control Tconv (comparison control Tconv versus GvHD Tconv) are shown. (J) Direct comparison of downregulated pathways in CD4^{+} T cells from CD40-AIR compared to control Treg treated mice. Significant enrichR comparison terms from Reactome database are plotted (adjusted pvalue < 0.05, manually selected pathways based on biological relevance are shown,

full list see Table S5). (K) vst transformed scaled gene expression values of genes from the Reactome pathway term Cell Cycle, Mitotic R−HSA−69278, identified in (J), were visualized across all samples using a heatmap. Selected genes significantly downregulated (adjusted pvalue < 0.05, Deseq2) in CD40-AIR Treg treated mice (comparison to Control Treg treated mice) are shown.

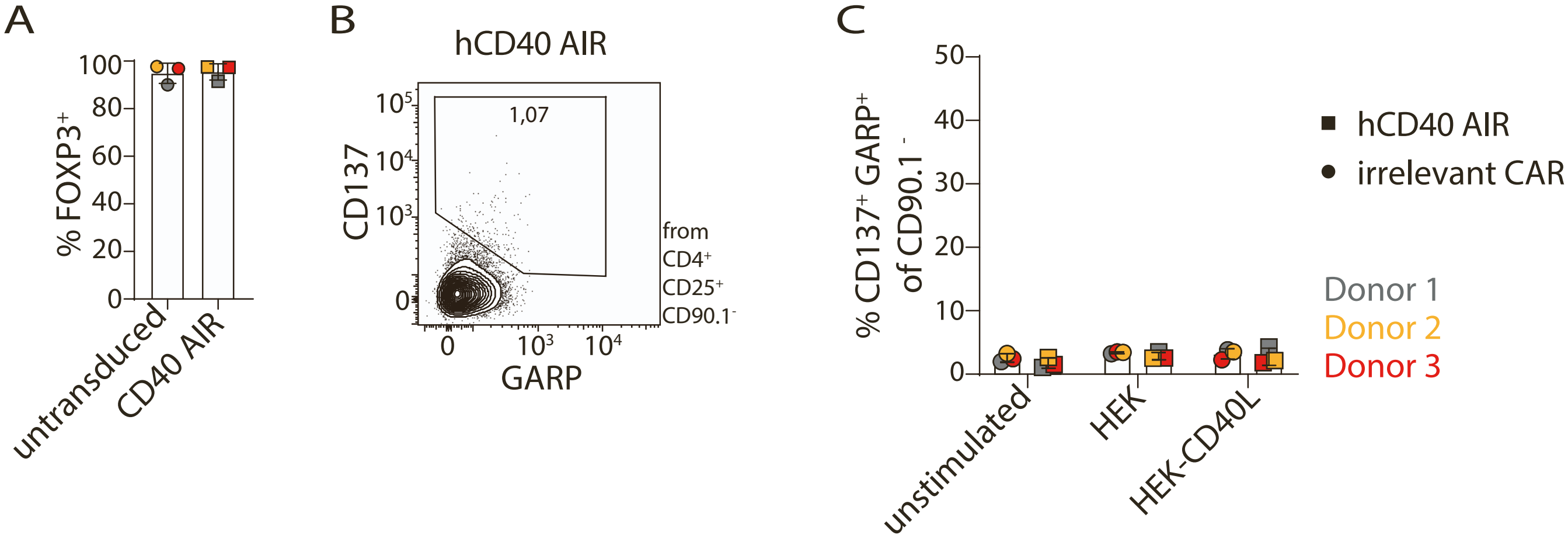
A
% FOXP3+
100
80
60
40
20
0
untransduced
CD40 AIR
B
hCD40 AIR
CD137
GARP
1,07
from
CD4+
CD25+
CD90.1-
C
% CD137+ GARP+ of CD90.1 -
50
40
30
20
10
0
unstimulated
HEK
HEK-CD40L
hCD40 AIR
irrelevant CAR
Donor 1
Donor 2
Donor 3

Figure S5. Human CD40-AIR expression and signaling.

(A) Purity of Treg cell cultures was assessed by intracellular staining for FOXP3 in expanded $CD4^+$ T cells with or without retroviral transduction 7 days after isolation. Summarized data for FOXP3 frequency for three different donors are shown. (B) Treg cells transduced with hCD40-AIR were analyzed via flow cytometry after 18 h without further stimulation. GARP and CD137 expression within the $CD90.1^-$ fraction of Treg cells (untransduced cells) from one representative donor is shown. (C) Treg cells transduced with hCD40-AIR or an irrelevant CAR (α-CEA, carcinoembrionic antigen) were analyzed via flow cytometry after 18 h without stimulation or in coculture with HEK cells or HEK cells expressing hCD40L protein on the surface. GARP and CD137 expression within the $CD90.1^-$ fraction of Treg cells (untransduced cells), summarized from three donors, are shown (n=3, two-way ANOVA). (D) Proliferation of CFSE labeled Tconv cells in the presence of CD40 AIR or CEA CAR Treg cells plotted as % of maximum proliferation of Tconv cells in the absence of Treg cells. Tconv cells were stained with CFSE and incubated with CD3/CD28 for 6 hours to upregulate CD40L expression. Cells were then co-cultured with CD40 AIR Treg cells or CEA CAR Treg cells for 7 days before proliferation as a function of CFSE dilution was measured by flow cytometry. Paired student's T-test; data derived from 2 independent experiments and 3 donors.

Supplemental Table Captions:

Table S1. CD40 AIR regulated genets

Table S2. HLA-A2 CAR regulated genes

Table S3. DE genes in control Tconv vs GvHD Tconv

Table S4. DE genes in CD40 Tconv vs GvHD Tconv

Table S5. DE genes in CD40 Tconv vs control Tconv

Table S6. Critical Commercial Assays

Lamina Propria Dissociation Kit	Miltenyi Biotec	Cat#130-097-410
GentleMACS C tube	Miltenyi Biotec	Cat#130-096-334
Anti-CD2 microbeads, human	Miltenyi Biotec	Cat#130-091-114
Anti-PE microbeads, ultrapure	Miltenyi Biotec	Cat#130-105-639
LS column	Miltenyi Biotec	Cat#130-042-401
MS column	Miltenyi Biotec	Cat#130-042-201
ACK lysis buffer	Gibco	Cat#A1049201
Foxp3 / Transcription Factor Buffer Set	eBiosciences	Cat#00-5523-00
RNEasy Plus Mini Kit	Quiagen	Cat#74134
round bottom 96 well plate	Corning	Cat#3799
Mouse T cell activation and expansion kit	Miltenyi Biotec	Cat#130-093-627
T Cell TransAct™, human	Miltenyi Biotec	Cat#130-111-160
CellTrace™ CFSE Cell Proliferation Kit	Thermo Fisher Scientific	Cat#V12883
Qiagen RNeasy Micro Kit	Qiagen	Cat#74004
High-Sensitivity RNA screentape	Agilent	Cat#5067-5579
SMART-seq Stranded Kit	Takara	Cat# 634444

Table S7. Chemicals, Plasmids and Recombinant Proteins

Recombinant human IL-2	Novartis	Proleukin S® 18 Mio U
TexMACS medium	Miltenyi Biotec	Cat#130-097-196
Proteinase K	Carl Roth	Cat#7528.5
HBSS	Gibco	Cat#14025092
PBS	Gibco	Cat#10010023
DPBS	Gibco	Cat#14190094
DMEM	Gibco	Cat#41965039
OptiMEM	ThermoFisherScientific	Cat#11058021
Bovine Serum Albumin	Sigma	Cat#A4503
DNase I	Roche	Cat#11284932001
TransIT-293	MoBiTec	Cat#Mir2700
Pancoll	PAN biotech	#P04-601000
Non Essential Amino Acid Solution (100x)	PAN biotech	#P08-32100
ß-Mercaptoethanol	PAN biotech	#P07-05100
MEM-Vitamin Sol. 100x	PAN biotech	#P08-41100
L-Glutamin (100x)	PAN biotech	#P07-05020
Natrium-Pyruvat 100mM	PAN-Biotech	#P04-43100
HEPES Buffer 1M	PAN biotech	#P05-01100
Penicillin-Streptomycin (10,000 U/mL)	Gibco	#15140122
pCL-Eco Retrovirus Packaging Vector	Novus Biologicals	NBP2-29540
pCL-Ampho Retrovirus Packaging Vector	Novus Biologicals	NBP2-29541
pMSCV-IRES-Thy1.1 DEST	Addgene (gift from Anjana Rao)	Cat#17442
recombinant murine CD40L	Peprotech	Cat#315-15

Table S8. Software and Algorithms

**Software**	**Manufacturer**	**Source**
Flowjo (V10.9.0)	BD Biosciences	(https://www.flowjo.com/solutions/flowjo/downloads/)
FACS Diva (V8.0.2)	BD Biosciences	(http://www.bdbiosciences.com/en-us/instruments/research-instruments/researchsoftware/flow-cytometry-acquisition/facsdiva-software)
GraphPad Prism (V9.5.0)	GraphPad Software Inc	(https://www.graphpad.com/)
SnapGene (V4.2.4)	GSL Biotech LLC	(https://www.snapgene.com/)
Biorender	Biorender	(https://www.biorender.com/)
